# A Myelin Debris Cleaner for Spinal Cord Injury Recovery: Polycaprolactone / Cell Membrane Assembled Scaffolds

**DOI:** 10.1002/advs.202503269

**Published:** 2025-06-26

**Authors:** Yuchen Zhou, Tao Xu, Yiyan Zhou, Nuo Chen, Zhengchao Wu, Zongze Yang, Changwei Yang, Xiaoqing Chen

**Affiliations:** ^1^ Department of Spine Surgery Affiliated Hospital of Nantong University Medical School of Nantong University Nantong 226001 China; ^2^ Department of Orthopedics Yancheng Dafeng People's Hospital Yancheng 224100 China; ^3^ School of Foreign Language Nanjing Normal University Nanjing 210024 China

**Keywords:** biomaterials, macrophage, myelin debris, PCL, spinal cord injury

## Abstract

After spinal cord injury (SCI), a mass of myelin debris derived from injured myelin sheath will be consistently generated and induce macrophages to be foam cells. It has been established that myelin debris and foam cells are negative on SCI recovery through direct and indirect neurotoxicity. Different from previous studies, the present research utilized efficient biological composite materials to adsorb myelin debris, exploring new avenues for solving foam cells and myelin debris following SCI. To achieve the strategy, the present author team has developed the biomaterial composed of polycaprolactone (PCL) nanofiber and pretreated macrophage membranes. Results in vitro and in vivo showed that the composite biomaterial effectively adsorbed myelin debris, with a result of few remaining foam cells, mitigated inflammation, minimal scarring, and favorable motor function recovery. Moreover, lipidomics and proteomics, from a metabolic perspective, further demonstrated the regulatory role of the composite biomaterial in myelin debris. Taken together, the composite biomaterial can effectively promote SCI recovery, which provides a novel insight for the treatment of SCI.

## Graphical abstract

pMM‐PCL, made up of pretreated macrophage menbrane and polycaprolactone, boasts the adsorption of harmful myelin debris in the microenvironment of spinal cord injury.

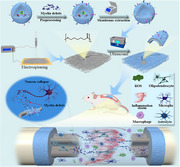

## 1 Introduction

After spinal cord injury (SCI), harmful debris, especially myelin debris, will continue to be produced at the injury site due to primary trauma and secondary injuries such as inflammation and oxidative stress, which seriously hinder the repair of SCI.^[^ ^1^, ^2^ ^]^ Based on our previous summary,^[^ ^3^ ^]^ myelin debris is mainly composed of lipid (e.g., cholesterol) and small amounts of protein (e.g., myelin associated inhibitors, MAIs). Accordingly, the obstruction of myelin debris to the repair of SCI can be attributed to two points: one obstruction is the collapse of axon growth cone directly owing to the MAIs (e.g., Nogo, Mag, Omgp, et al.); the other is the deterioration of the microenvironment indirectly mediated by lipid. After SCI, the body will protectively remove these harmful debris by attracting phagocytes, such as Bone marrow derived macrophages (BMDMs), Microglia, Oligodendrocytes, and Astrocytes, et al. to relieve their direct damage. However, though the lipid‐rich myelin debris gets cleared and then the microenvironment appears to be improved, phagocytes tend to change their state after phagocytosis.^[^ ^4^ ^]^ For example, most BMDMs will be changed into “foam cells” irreversibly due to the excessive accumulation of intracellular lipid and then remain in the center of injured spinal cord for a long time. What's worse, these foamy macrophages are always induced to be more pro‐inflammatory and more biased toward scar promoting, which is adverse to SCI recovery. Therefore, the removal of myelin debris and foam cells will make a great contribution to the repair of SCI.

Nevertheless, there have remained controversial debates on the priority for dealing with myelin debris and foam cells. On one hand, scholars aim to treat the direct obstruction of myelin debris to rescue axon growth cones. For example, they have succeeded in relieving the contact inhibition of axon regeneration through blocking receptors of myelin debris on neurons (e.g., Ngr) or promoting phagocytes’ engulfment to myelin debris.^[^ ^5^, ^6^ ^]^ On the other hand, other scholars have a primary goal of removing foam cells. They tend to block phagocytic receptors to myelin debris to impede the transformation of macrophages into foam cells.^[^ ^7^ ^]^ Though the twin approaches mentioned above can be somehow successful, they often only address one aspect of the harm caused by myelin debris. There is no explanation on how to handle the foam cells in the first approach, nor how to address the myelin debris next in the latter. Therefore, it is essential and challenging to develop a strategy that can simultaneously reduce myelin debris and foam cells.

The strategy, aimed at myelin debris and foam cells, has been explored previously. Researchers tried to solve foam cells after myelin debris had been cleared by macrophages and they found that, compared with initial macrophages, lipid autophagy (lipophagy) in foam cells was impaired significantly, resulting in the inability of endocytosed lipids to get metabolized.^[^ ^8^ ^]^ Therefore, after enhancing the activity of lipophagy, they successfully reduced foam cells, which improved their proinflammatory and scar‐promoting effect.^[^ ^9^ ^]^ Though this approach reduced not only myelin debris but also foam cells, there existed a time interval between the formation and reduction of foam cells when foam cells had a chance to deteriorate the microenvironment.

Combining these existing explorations, our team has developed a radically new strategy: myelin debris can be removed away from the spinal cord by the special biological materials, and “detain” these myelin debris outside. In this way, the direct neurotoxicity of myelin debris can be alleviated, and foams cells can be reduced simultaneously. Thereupon, the well‐designed composite biomaterial is adopted, whose superiority lies in its ability to adsorb myelin debris and immobilize it to the outside site of injured spinal cord.

To achieve the adsorption of myelin debris, our team finally utilized the specific binding of the surface receptors of macrophage cell membranes to myelin debris from a multidisciplinary perspective. Compared to intact cells, cell membrane retains the original biological activity without the risk of immunological rejection, contributing to their widespread use in nanomedicine, particularly the “core‐shell” structure composed of nanoparticle core and cell membrane shell.^[^ ^10^, ^11^ ^]^ The “shell” composed of the cell membrane can help the core nanoparticles achieve the targeted release and evade the clearance by the immune system, which prolongs their circulation in the body. However, the application mode of cell membrane should not be limited to the core‐shell structure of nanoparticles, and the application purpose should not be limited to the “targeting” and “immune evasion”. Therefore, Chen et al. have begun to warp cell membranes on electrospinning to increase its affinity to cells, and Gao et al. innovatively achieved the clearance of inflammatory factors, viral particles and other harmful substances by membrane receptors’ adsorption.^[^ ^12^, ^13^, ^14^ ^]^ Overall, the binding ability of membrane receptors to their ligands is promising for application. In addition, it has been shown that membrane receptors mediate the recognition and phagocytosis of myelin debris.^[^ ^15^ ^]^ Taking these into account, our team decides to obtain the membrane of BMDMs as the core part of biomaterial to adsorb myelin debris.

After cell membrane was picked to adsorb myelin debris, the adsorption efficiency was further considered to optimize the whole material. It has been found that the expression of receptors that bind to myelin debris on macrophage membranes is upregulated in SCI microenvironment.^[^ ^16^ ^]^ Therefore, we pretreated macrophages with spinal cord homogenate before extracting their membranes, so that the obtained cell membranes boast a greater number of target receptors.

The poor physical properties of cell membranes lead to its failure of working steadily at the injury site, and thus a suitable carrier is expected. After combining the current technology in materials, our team decided to use polycaprolactone (PCL) nanofiber scaffold prepared by electrospinning equipment. In the field of biomedicine, electrospinning scaffolds are widely used as carriers for slow release of drugs owing to the high simulation of extracellular matrix, excellent mechanical stability, and satisfying fiber continuity.^[^ ^17^, ^18^ ^]^ However, for the diversity of raw materials and the complexity of morphological parameters, electrospinning must meet the following basic requirements before the application in nerve regeneration: proper biocompatibility, suitable biodegradability, and qualified mechanical characteristics.^[^ ^19^ ^]^ Therefore, PCL was finally chosen as the raw material.^[^ ^20^, ^21^, ^22^ ^]^ In addition, other parameters of electrospinning scaffold, such as fiber diameter, pore size, wetness, and roughness, are carefully considered before electrospinning is to be applied.

**Figure 1:**
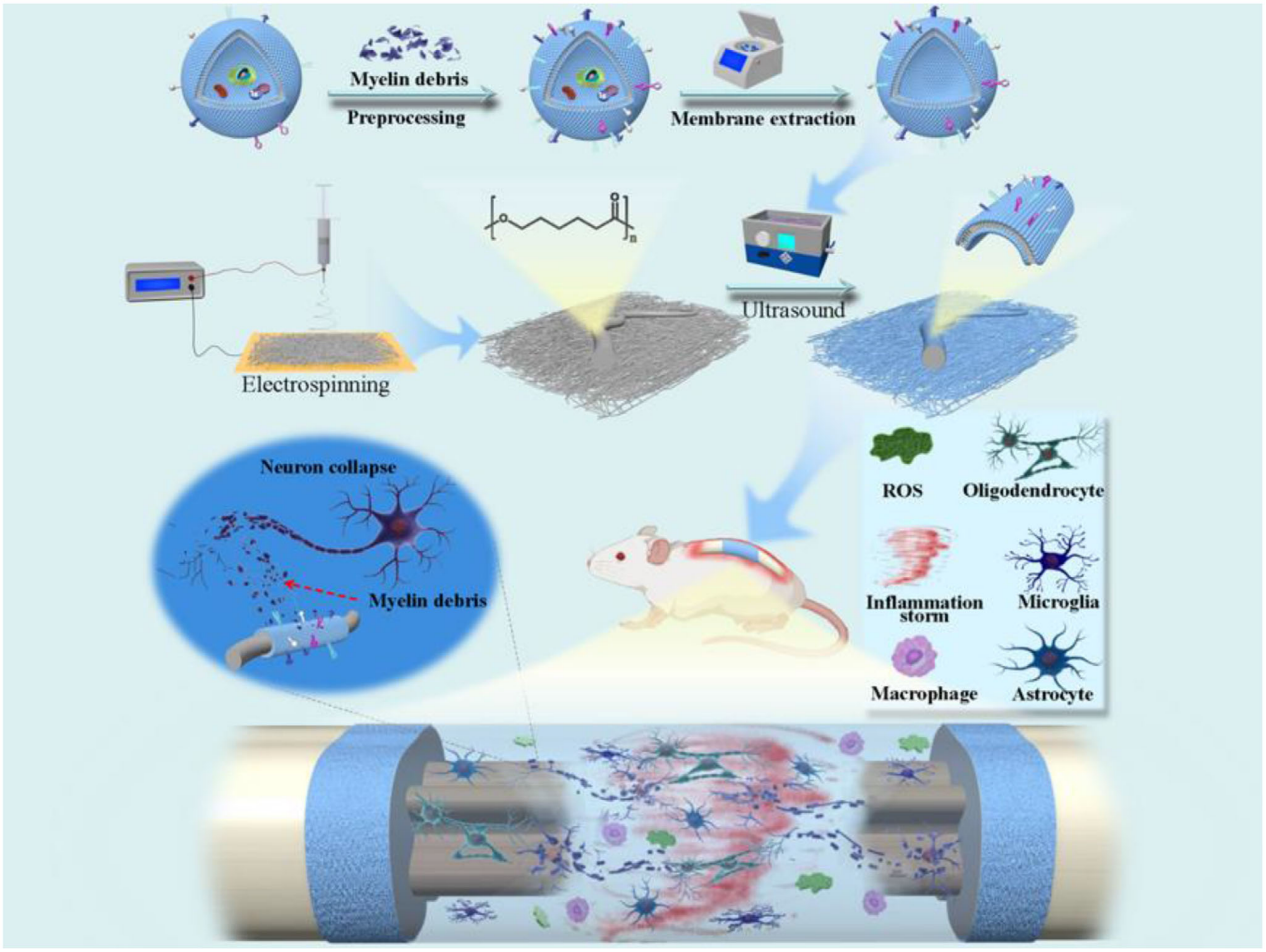
Graphical abstract of the synthesis of pMM‐PCL and its regulation on the pathological process of SCI.

Herein, we first explore the strategy of “myelin debris adsorption” by pMM‐PCL to promote SCI repair, which simultaneously reduces myelin debris and foam cells. The results demonstrated that we have achieved the adsorption of myelin debris, the reduction of foam cells, and the regulation of the microenvironment by pMM‐PCL. Meanwhile, the alleviation of neural inhibition, the relief of scar, the enhancement of neural regeneration, and the recovery of motor function were also achieved in the spinal cord contusion model applied with pMM‐PCL. In general, our strategy and the coordinated materials alleviated the damage of myelin debris and foam cells both in vitro and in vivo, which provided a new perspective for SCI recovery (**Figure** **1**).

## 2 Experimental Section

### 2.1 Chemical Reagents and Antibodies

In vitro and in vivo experiments: Raw 264.7, PC12, and fetal bovine serum (FBS) were purchased from Wuhan Pricella Biotechnology. Dulbecco's modified eagle medium (DMEM), Ham's F12 nutrient medium, Penicillin‐Streptomycin, Phosphate Buffered Saline (PBS), and Phenylmethanesulfonylfluoride (PMSF) were purchased from Thermo Fisher, USA. Radio‐Immunoprecipitation Assay Lysis buffer (RIPA) was purchased from Solaibao Technology, China. Modified Oil Red O Staining Kit was purchased from Beyotime Biotech Inc. (Beyotime, China). CD36 Polyclonal antibody, Myelin basic protein (MBP) polyclonal antibodies, CD206 polyclonal antibodies, INOS polyclonal antibodies, CD86 polyclonal antibodies, Arg1 polyclonal antibodies, MAP2 polyclonal antibodies, GFAP polyclonal antibodies, TUBB3 polyclonal antibodies, and NF200 polyclonal antibodies were purchased from Proteintech, USA.

In omics analysis: listed in Supplementary Material S2 (Supporting Information).

### 2.2 Synthesis of pMM‐PCL

#### 2.2.1 Acquisition of Spinal Cord Homogenate

After ethical approval obtained, Sprague‐Dawley (SD) rats aged 8–10 weeks were sacrificed at 3‐days post injury (3‐dpi). Then the spinal cord was removed, and the spinal cord membrane was stripped. The obtained spinal cord specimens were temporarily stored in ‐80 °C. On the day of macrophage processing, spinal cord specimens were homogenized with a homogenizer after adding an appropriate amount of PBS, resulting in a final concentration of 100 mg mL^−1^ of homogenate. When stimulating macrophages, original homogenate was added to each well and the final stimulation concentration was 10 mg mL^−1^.

#### 2.2.2 Extraction of Myelin Debris

Based on the summary of previous methods, sucrose gradient centrifugation (0.83 M and 0.32 M) was finally decided to obtain myelin debris.^[^ ^23^ ^]^ The endotoxin of obtained myelin debris was below the detection limit of Limulus Amebocyte Lysate assay. Myelin debris was used at a concentration of 100 mg mL^−1^. Specific steps are listed in Supplementary Material S4 (Supporting Information).

#### 2.2.3 Extraction of Membranes of Pretreated Macrophages

To obtain the pretreated macrophages, spinal cord homogenate (10 mg mL^−1^) was co‐cultured with Raw 264.7 for 48 h. Pretreated macrophages (5×10^7^ cells) were collected and suspended in 3 mL of hypotonic lysis buffer containing RIPA and 10% (v/v%) PMSF. The macrophage suspension was subjected to an ice bath for 30 min and then freeze‐thawed for 3–5 times (frozen in ‐80° C, thawed in 37 ° C). Then the treated cell suspension was centrifuged at 20 000 g for 25 min at 4 ° C to remove the precipitated intracellular components. The supernatant was collected and centrifuged at 150 000 g for 35 min at 4 ° C. The final precipitate collected was purified pretreated macrophage membrane (pMM). The precipitate was added to PBS solution and gently resuspended, stored at ‐80 ° C.

#### 2.2.4 Synthesis of PCL Nanofiber

PCL with a molecular weight of 4.5w, 6w, 8w, or 10w was dissolved in hexafluoroisopropyl alcohol (HFIP) (Sigma Aldrich, USA) at concentrations of 8%, 10%, 12%, or 15%, respectively, and thoroughly mixed until the solution was uniform. The solution was loaded into a syringe (Affiliated Hospital of Nantong University, China) and passed through a blunt stainless‐steel needle (inner diameter = 0.8 mm; Outer diameter = 2.0 mm). The droplet was stretched onto the fiber at a constant flow rate of 1.2 mL h^−1^ at 18 kV (VC100, Glass Mann, Japan). A distance of 18 cm was maintained between the tip and the collector, and room temperature was maintained at 28 ° C and humidity at 45% throughout the procedure. The finished product was dried in an oven (Xinrui Company, Nantong, China) overnight to stabilize the structure.

#### 2.2.5 Synthesis of pMM‐PCL

The collected electrospinning fibers are placed into an appropriate amount of cell membrane suspension to ensure that the fibers are completely immersed. The container filled with the fibers and the cell membrane suspension is then placed in an ultrasonic cleaner. The ultrasonic power is set to 4000 hz, the ultrasonic time is set to 30–60 min, and the ultrasonic temperature is precisely controlled at 25–35 °C. During the ultrasonic process, the energy of ultrasound promotes the cell membranes to fully contact and combine with the fiber surface, forming a uniform cell membrane coating. After the ultrasonic treatment is completed, the fibers are carefully taken out, gently rinsed with deionized water to remove the unbound cell membranes on the surface, and then dried at room temperature or in a vacuum drying oven.

### 2.3 Characterization and Physical Properties

#### 2.3.1 Scanning Electron Microscopy (SEM) Analysis

SEM was used to observe the characterization of composite materials. The composite material was first cut into 1.2 cm ×  1.2 cm films, and the surface impurities were cleaned with high‐pressure nitrogen gas, and then fixed on the sample table. The immobilized material was dehydrated and dried. The surface of the sample was sprayed gold (20 mA, 90 s) through a spray plating machine and observed under S‐5000 SEM (Guoyi Electronics, China).

#### 2.3.2 Identification of Mechanical Properties

Two experimental groups were set up: the PCL group and the pMM‐PCL group. For each group, a minimum of three independent samples, each 50 mm in length and 10 mm in width, were chosen. The test was carried out at a temperature of 25 °C and an ambient humidity of 35%. Initially, the sample was fastened using a metal frame, and the clamping distance of the testing machine was adjusted to 30 mm. Subsequently, the test commenced with a head velocity of 10 mm min^−1^, an initial tensile force of 6 mN, and a step‐by‐step applied force gradient of 0.50 mN min^−1^. The test was terminated once the sample fractured. Stress‐strain curves were recorded for every sample, and their fracture strain values were computed. The entire test was conducted using a WSD series tensile testing machine (manufactured by Wanchen, China).

#### 2.3.3 X‐Ray Photoelectron Spectroscopy (XPS) Analysis

The experimental groups were the same as above. The two groups of samples were cut into an area of 5 mm ×  5 mm and kept as dry as possible to facilitate vacuum sampling. Due to the weak conductivity of the material, charge calibration is required before analysis. Finally, the elements present on the surface of the material can be determined from their specific photoelectron binding energy. The whole experiment was performed on a Kratos Axis Ultra DLD multifunction electron spectrometer.

#### 2.3.4 Degradation of pMM‐PCL

The experimental groups were identical to those described previously. For each group, a minimum of 3 randomly chosen samples were selected to measure the degradation rate of pMM‐PCL. Each sample had a weight of no less than 150 mg. Before being subcutaneously implanted into SCI‐induced SD rats, the initial mass (W0) of each material was carefully recorded. Then, at 0, 1, 3, 5, 7, 14, 21, 28, 35, 42, and 49 days after the implantation, the residual mass (W1) was measured and noted. The degradation rate of the material was calculated according to the formula: [(W0−W1)/W0] ×100%. The experiment was carried out in the standard animal‐rearing environment of the Animal Experimental Center at Nantong University. All the experimental animals were mature SD rats with a moderate body weight of around 250 g. The rats selected for the measurements were chosen randomly and had no skin‐related diseases.

### 2.4 In Vitro Experiment

#### 2.4.1 Adsorption of Myelin Debris

To cell membrane: the extracted cell membrane (from macrophages and pretreated macrophages) was fixed in the middle of bisected discs in a rectangular shape in 24 well plate and cultured with myelin debris (200 µl) and F12 medium (10 mL) for 24 h. The residual myelin debris was sucked up, and MBP polyclonal antibody was added after 5 times of PBS washing. The shape and density of fluorescence were observed under the microscope to test the adsorption capacity of cell membrane to myelin debris in 2 groups. To material: four experimental groups were set up, two of them were added with PCL and pMM‐PCL placed in a circle around the edge of the petri dish respectively, and the last two were added with slides and pMM‐loaded slides around the edge of the petri dish respectively. After adding myelin debris and Raw264.7, the numbers of foam cells (expressed by ORO staining) and thus the circumstance of microenvironment (expressed by immunofluorescence staining) in each group were used to reflect the adsorption of myelin debris in four groups after 48 h.

#### 2.4.2 Coculture of pMM‐PCL and Neurons

To observe the support and nutritional effects of composite nanofiber scaffolds on neurons, neurons extracted from SD rats aged 0–3 days were implanted on cell culture plates coated with polylysine (10 mg mL^−1^), some of which with pMM‐PCL scaffold covered, and others with conditioned medium only, differences in the activity of neurons in the two groups were observed.

Neuron extraction: Newborn SD rats within 24 h were cleaned with 75% ethanol, and then anesthetized, decapitated, and placed in a beaker containing DMEM (DMEM should not cover the head). Prepare three Petri dishes placed on ice, and the same amount of high‐glucose DMEM was added respectively. The severed heads were put into No. 1 Petri dish to peel off the brain, and the surface leptomeninges of the brain were separated one by one in No. 2 Petri dish. After dissection, the cerebral cortex/hippocampus was extracted with a curved forceps and placed into a No.3 Petri dish. The tissue was digested with 0.25% papain for 20 min (shaking the Petri dish every 5 min), and the digestion was stopped with DMEM containing 10% fetal bovine serum. The digested tissue was gently blown, and the cell suspension was adsorbed and filtered with a 100‐mesh filter on a 50 mL centrifuge tube, then the cell suspension was transferred into a 15 mL centrifuge tube and centrifuged for 5 min at 1000 r min^−1^. After the cell was re‐suspended, the cells were incubated on a 6‐well plate coated with poly‐L‐lysine. After the cells were fully attached and grew, IF staining with neuron‐specific antibodies was performed to verify the purification rate of neurons.

#### 2.4.3 Cell Proliferation of PC12

The experimental groups were Control, pMM, PCL, and pMM‐PCL. PC12 was implanted in a 6‐well plate (5 × 10^5^ per well). After PC12 fully filled the plate, 3 parallel scratch holes were made on each plate with the tip of 100 µL microtubule and then myelin debris was added in each plate. Random wells were added with pMM‐PCL, and others were added with medium, PCL, and pMM loaded randomly. After 24 h, the wound closure was quantified by measuring the remaining unmigrated area with ImageJ. The migration ability was expressed by the percentage of the closure of gap distance. Cells were imaged by Zeiss AxioCam microscope, and each group of experiments should be conducted at least 3 times.

#### 2.4.4 Invasion of Macrophages

The experimental groups were Control, pMM, PCL and pMM‐PCL. The invasion and migration levels of macrophages were evaluated in a 24‐well plate with Transwell chambers of 8 µm pore size and 6.5‐mm diameter. First, macrophages (3 × 10^4^ per well) were added in 200 µL FBS‐free medium and then placed in the upper chamber covered with Matrigel (80 µL; dilution 1:4; Sigma, St Louis, MO). Following the medium with 10% FBS was added in the lower chamber, the slides (Control group), pMM‐loaded slides (pMM group), pMM‐PCL (pMM‐PCL group), and PCL (PCL group) were respectively placed in the edge of lower chamber after their co‐culture with myelin debris for 24 h and washing with PBS for 3 cycles. All plates were incubated at 37 °C for 1d, and the number of macrophages in the lower chamber was used to reflect the attraction of myelin debris‐loaded pMM‐PCL to macrophages (illustrated in Supplementary Material S6, Supporting Information).

#### 2.4.5 Oil Red O (ORO) Staining

ORO staining was performed to label foam cells, reflecting the BMDMs engulfing myelin debris after SCI or in vitro.^[^ ^24^ ^]^ For cell experiments, RAW 264.7 cells were treated with spinal cord homogenate (10 mg mL^−1^) for 48 h to obtain foam cells. Cells were first washed three times with PBS and fixed with formaldehyde for 30 min. Then the fixative was discarded, and the cells were washed three times with distilled water and stained with ORO staining solution (C0158S1, Beyotime, China) for 60 min. The staining solution was discarded, and the cells were washed with the deionized water. For tissue experiments, frozen sections of the spinal cord were dried and washed with PBS, stained with ORO staining solution for 10 min, treated with 60% isopropanol for 5 min, and finally washed with distilled water.

### 2.5 In Vivo Experiment

#### 2.5.1 Animal Ethics

The experimental animals in this research were all female SD rats (weighing 250 g, and aged 8w), which were obtained from the Experimental Animal Center of Nantong University. SD rats were raised in strictly controlled humanitarian environment with light and dark cycling for 24 h (the light‐dark ratio is the same). Experimental rats had free access to sufficient water and food.

To simulate common clinical SCI cases better, the spinal cord contusion model of T10 was selected.^[^ ^25^ ^]^ Before surgery, female SD rats were anesthetized by intraperitoneal injection of 1% sodium pentobarbital (50 mg kg^−1^). Next, the hair on the back was shaved and sterilized with iodine. After that, a small incision was made parallel to the long axis, the skin and subcutaneous fascia were cut, the muscles were bluntly separated, and the lamina was carefully uncovered to expose the T10 segment of the spinal cord. Allen percussion was used to hit the surface of the T10 spinal cord segment, and the rat's bilateral hind limbs were completely relaxed after convulsing, indicating that the spinal cord contusion model was successful. A total of 60 SD rats were randomly divided into 5 groups. The Sham group was only treated with laminectomy, the SCI group was only treated with spinal cord contusion, and the MM, PCL, and MM‐PCL groups were covered with their own materials on the surface of the contused spinal cord, and the dura mater of each group was opened. Regarding the material size, we finally decided to use pMM‐PCL with a size of 10 mm × 5 mm for wound coverage based on existing studies on the extent of myelin debris disintegration after SCI (illustrated in Supplementary Material S3, Supporting Information).^[^ ^26^, ^27^ ^]^ After surgery, each rat was injected with antibiotics and buprenorphine to prevent infection and reduce pain, and the bladder was artificially pressed per 8 h to assist urination until urination function was restored. Finally, samples were taken at 6w, and the spinal cord was carefully denuded.

#### 2.5.2 Catwalk and Basso, Beattie, and Bresnahan (BBB) Score

To evaluate the functional recovery of rats after SCI, catwalk, and BBB score were performed. Operators and recording personnel were unaware of the grouping of SD rats; each group of SD rats was treated independently by three randomly assigned team members, and there was no communication the whole time. Catwalk: The rat was placed on one end of the walking stage and allowed to walk freely to the other end. The high‐speed camera recorded the walking process of the rats, and the system automatically extracted the footprint information. VisuGait software was used to analyze the footprint images, and the gait parameters of the rats were automatically identified and analyzed, such as walking cycle, support distance, support duration, swing duration, braking duration, and propulsion duration. BBB score: The BBB score ranges from 0 to 10, and the movement of both hind limbs is scored at 0, 1, 3, 7, 14, 21, 28, 35, and 42‐dpi. Each score is the average of three independent outcomes.

#### 2.5.3 Histology

After euthanasia, the rats were intraperitoneally perfused with 0.9% saline and then fixed with 4% paraformaldehyde. The spinal cord containing the injury site was carefully removed and placed in 4% paraformaldehyde for 24 h. After dehydrated, the removed spinal cord segments were encapsulated in OCT and processed with a cold microtome (Leica‐cm1950, Clinical Experimental Center, Affiliated Hospital of Nantong University) to obtain coronal slices with thickness of 8 to 12 µm.

#### 2.5.4 Masson Staining

Staining was first performed with the prepared Weigert iron hematoxylin staining solution for 5–10 min. Then the acidic ethanol differentiation solution was used for 5–15s and washed off with water. Next, the spinal cord was treated with Masson blue solution for 3–5 min and washed off with distilled water for 1 min. Ponceau fuchsin staining solution was used further for 5–10 min. After being treated with weak acid working solution for 1 min, the spinal cord was washed with phosphamolybdic acid solution for 1–2 min and with weak acid working solution for 1 min again. The spinal cord was directly placed into aniline blue staining solution for 1–2 min and washed with weak acid working solution for 1 min. Finally, the spinal cord was dehydrated with different concentrations of ethanol, transparent with xylene 3 times (each for 1–2 min), and sealed with neutral gum. Each stained spinal cord sample was photographed with a light microscope (Leica dm 3000) and analyzed using Image J (National Institutes of Health) software. All samples were observed and photographed under a Keyence fluorescence microscope.

#### 2.5.5 Immunofluorescence Staining

Frozen sections were thawed at RT, washed three times with PBS, and blocked with blocking solution (5% goat serum) for 30 min at RT, followed by permeabilization with 0.1% TritonX‐100 for 15 min. The primary antibody was added, incubated at 4 °C for 16 h, and the secondary antibody was added after 3 times of PBS washing (the subsequent operations should be carried out in dark), incubated at RT for 2 h, and fully washed with PBS for 3 times. The nuclei were stained with 4',6‐diamidino‐2‐phenylindole (DAPI). Finally, the cover glass was sealed with an anti‐fluorescence quencher.

#### 2.5.6 Omics Analysis

Details are provided in Supplementary Material S2 (Supporting Information).

#### 2.5.7 Statistical Analysis

In this study, each group of experiments was carried out at least 3 times. All statistical data were analyzed by GraphPad Prism version 8.0 (San Diego, USA). For the sample calculation, the statistical power was set at 0.90, the significance level was set at 0.05, and P <0.05 was considered statistically significant. The data of each group were calculated as mean ± standard deviation (SD). Student's t test was used for comparison between two groups, and analysis of variance (ANOVA) followed by SNK‐q test was used for statistical differences between multiple groups. BBB scores were analyzed by two‐way repeated measures analysis of variance.

## 3 Results

### 3.1 Preparation of pMM

**Figure 2:**
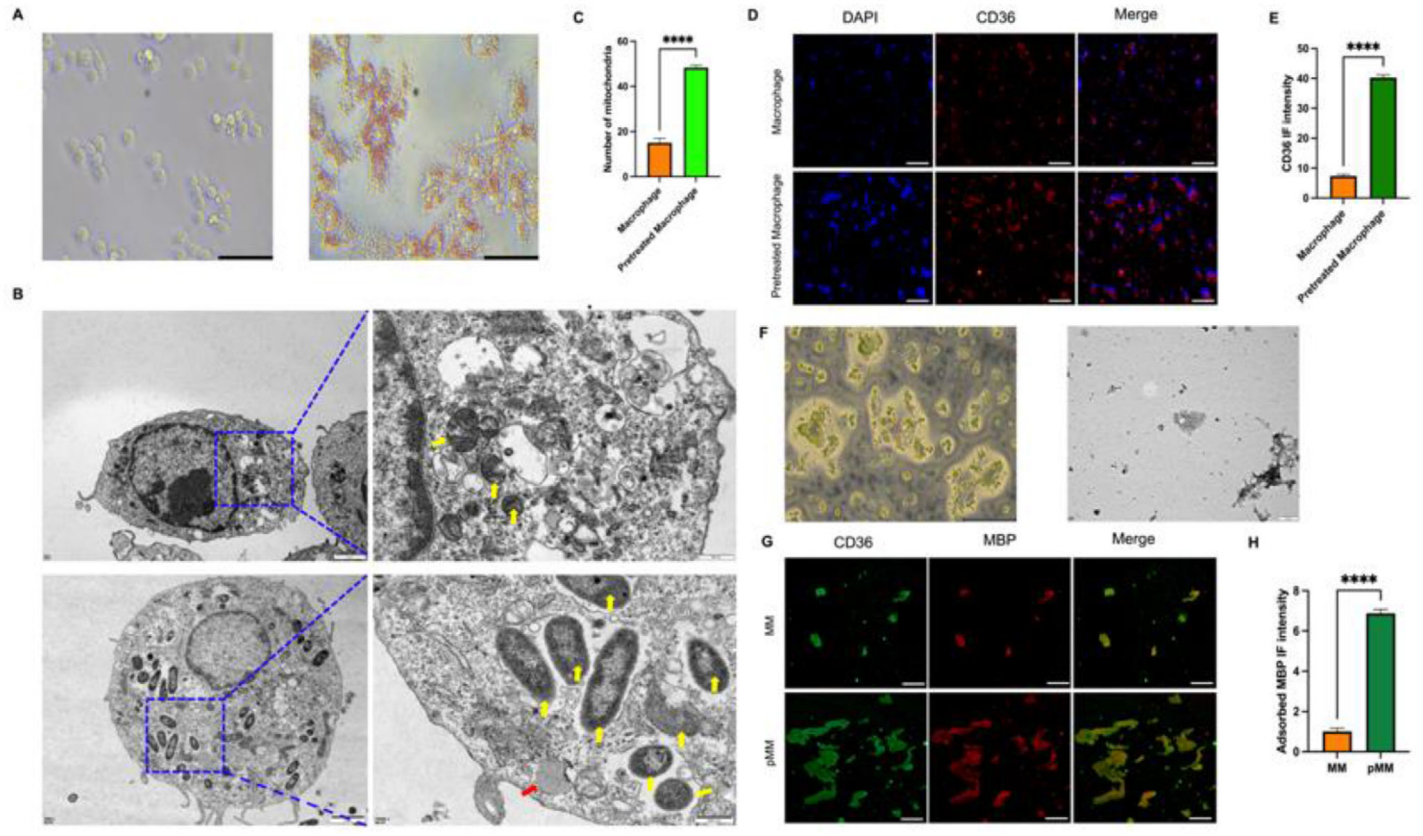
Preparation of pMM and validation of its adsorption to myelin debris. A) Optical microscope comparison of macrophages and macrophages pretreated with myelin debris. Scale bar: 200 µm (× 10). B) Transmission Electron Microscope (TEM) comparison of macrophages and macrophages pretreated with myelin debris. Scale bar: 2 µm (× 200). The yellow in the picture referred to mitochondria and the red referred to lipid droplets. Scale bar: 500 nm (× 800). C) Statistical analysis of the number of mitochondria (n = 3), assessed via t‐test. D) Comparison of the number of CD36 receptors on the surface of macrophages and macrophages pretreated with myelin debris. Scale bar: 100 µm (× 5). E) Statistical analysis of CD36 IF intensity (n = 3), assessed via t‐test. F) Optical microscope and TEM of pretreated macrophage cell membrane. Scale bar: 200 µm (× 5). G) Comparison of the adsorption efficiency to myelin debris between macrophage membrane and pretreatment macrophage membrane. Scale bar: 100 µm (×5). H) Statistical analysis of adsorbed MBP IF intensity (n = 3), assessed via t‐test. ****p < 0.05.

To verify the quantitative changes of phagocytic receptors in macrophages after the stimulation of myelin debris, we performed the experiment by making use of the representative relevant receptor CD36.^[^ ^15^ ^]^ As is shown in **Figure** **2**A‐B, the macrophages became larger after engulfing myelin debris and appeared to be “foam‐like”, when many lipid droplets could obviously be found in these cells, reflecting the successful stimulation of macrophages by myelin debris (namely foam cells). Moreover, as is depicted in previous studies, phagocytoreceptor expression on the surface of macrophages was substantially enhanced after the myelin debris stimulation, which was confirmed by the clearly enhanced fluorescence density of CD36 in Figure 2D‐E (from 7.35 ± 0.56 to 40.24 ± 0.93). Subsequently, the membrane from pretreated macrophages (pMM) and macrophage (MM) was extracted by differential centrifugation. Then, some assays were performed to verify the successful extraction of the cell membrane. Under the optical microscope, multiple fragmented structures could be observed as a single cell aggregation, and similar manifestations could be found under electron microscope (Figure 2F). All these results indicated that cell membrane was successfully obtained. Next, the most critical step is to verify that the obtained cell membrane has the ability to bond and withhold myelin debris. Therefore, the obtained MM and pMM were individually fixed in the well plate. Then, myelin debris was added, and the mixtures were co‐cultured for 24 h. After thoroughly washing away the remaining free myelin debris, we observed the immobilized myelin debris on the cell membranes. The fluorescence results presented in Figure 2G‐H showed that myelin debris had been adsorbed by the obtained cell membranes. Notably, the number of fixed myelin debris in the pMM group (6.87 ± 0.19) was significantly higher than that in the MM group (1.01 ± 0.19), which could be attributed to the increased expression of binding receptors in the pMM group. Therefore, we have successfully demonstrated that the obtained pMM from pretreated macrophages could efficiently adsorb myelin debris.

### 3.2 Preparation of PCL

**Figure 3:**
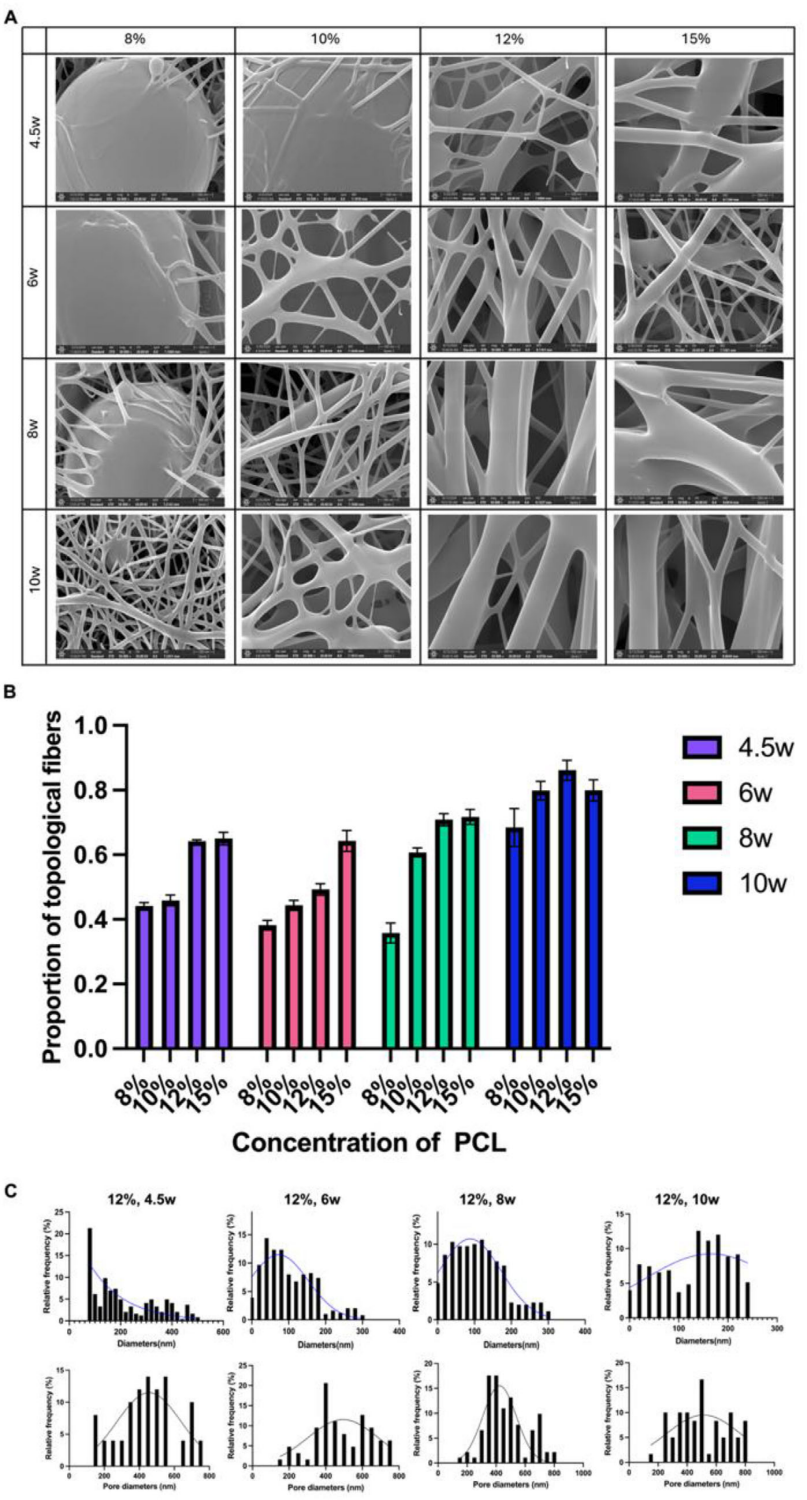
Preparation of PCL electrospinning. A) 16 kinds of PCL electrospinning with different mass fractions and molecular weights. Scale bar: 500 nm (× 50 000). B) Proportion of electrospinning without beads under each synthesis condition (n = 3). C) Size distribution of pore size and diameter of electrospinning with 12% mass fraction (n = 3), assessed via Nonlinear regression (Gaussian).

After PCL was ascertained as the raw material of nanofiber, the appropriate concentration and molecular weight were further explored under the reference of previous studies.^[^ ^28^, ^29^ ^]^ As is shown in **Figure** **3**A, four mass fractions of 8%, 10%, 12%, 15%, and four molecular weights of 4.5w, 6w, 8w, and 10w were set with pairwise combinations, resulting in a total of 16 kinds of topological electrospinning. Despite different mass fractions and molecular weights, there is no significant difference in the hydrophilicity and biocompatibility of the electrospinning prepared by the same material—PCL, which has hydrophobic ester bond (‐coo‐).^[^ ^30^ ^]^ Thereupon, the decision on the most suitable one among 16 kinds of electrospinning was based on the comprehensive consideration about the number of beads, diameter size and pore size. The previous research has shown that the number of beads in electrospinning goes hand in hand with the degradation rate. Furthermore, the electrospinning with a larger diameter and pore size enables the macrophages in contact with it to be more prone to differentiate into an anti‐inflammatory phenotype. This effectively mitigates the potential side effects of the material itself.^[^ ^31^, ^32^, ^33^ ^]^ According to our experimental results, the diameter and pore size of the electrospinning were proportional to the PCL mass fraction. Therefore, four kinds of electrospinning at 15% mass fraction should have been further considered for both large diameters and pore sizes. However, we found that the PCL solution at this mass fraction had high viscosity, large surface tension and strong intermolecular adhesion, under which condition diameter of these electrospinning was always uneven and blockage phenomenon often occurred during the spinning process. It can be seen in Figure 3 that the effective fiber (with few beads) ratios at 4.5w, 6w, 8w, and 10w were 65.01% ± 1.95%, 64.29% ± 3.24%, 71.71% ± 2.35%, and 79.93% ± 3.28% respectively. As a result, the mass fraction of 15% was excluded. In addition, the electrospinning at 8% and 10% mass fraction was also not selected because its low spinning viscosity always resulted in disordered fibers and obvious bead structure. As is shown in Figure 3A‐B, the effective fiber ratios at the molecular of 4.5w, 6w, 8w, and 10w and mass fraction of 8% were 44.10% ± 1.11%, 38.19% ± 1.49%, 35.80% ± 3.10%, and 68.43% ± 5.88% respectively, and the effective fiber ratios at the molecular of 4.5w, 6w, 8w, and 10w and mass fraction of 10% were 45.88% ± 1.68%, 44.37% ± 1.54%, 60.70% ± 1.45%, and 79.84% ± 2.84% respectively. Thereupon, PCL at the mass fraction of 12% was further considered, whose effective fiber ratios at the molecular of 4.5w, 6w, 8w, and 10w were 54.19% ± 0.44%, 49.25% ± 1.81%, 70.84% ± 1.88%, and 86.11% ± 3.05% respectively, diameters at the molecular of 4.5w, 6w, 8w, and 10w were 100.77 ± 69.15, 215.60 ± 125.71, 300.86 ± 83.44, and 378.87 ± 90.46 respectively, and pores at the molecular of 4.5w, 6w, 8w, and 10w were 453.6 ± 161.43, 483.42 ± 152.89, 470.92 ± 148.08, and 503.6 ± 172.46 respectively (Figure 3B‐D). Eventually, PCL at the mass fraction of 12% and molecular of 8w was chosen as the follow‐up experimental material, which exhibits a relatively uniform morphology, a small number of beads, and high proportion of large diameter and pores, meeting the requirements for excellent biocompatibility and favorable biodegradability.

### 3.3 Preparation and Characterization of pMM‐PCL

**Figure 4:**
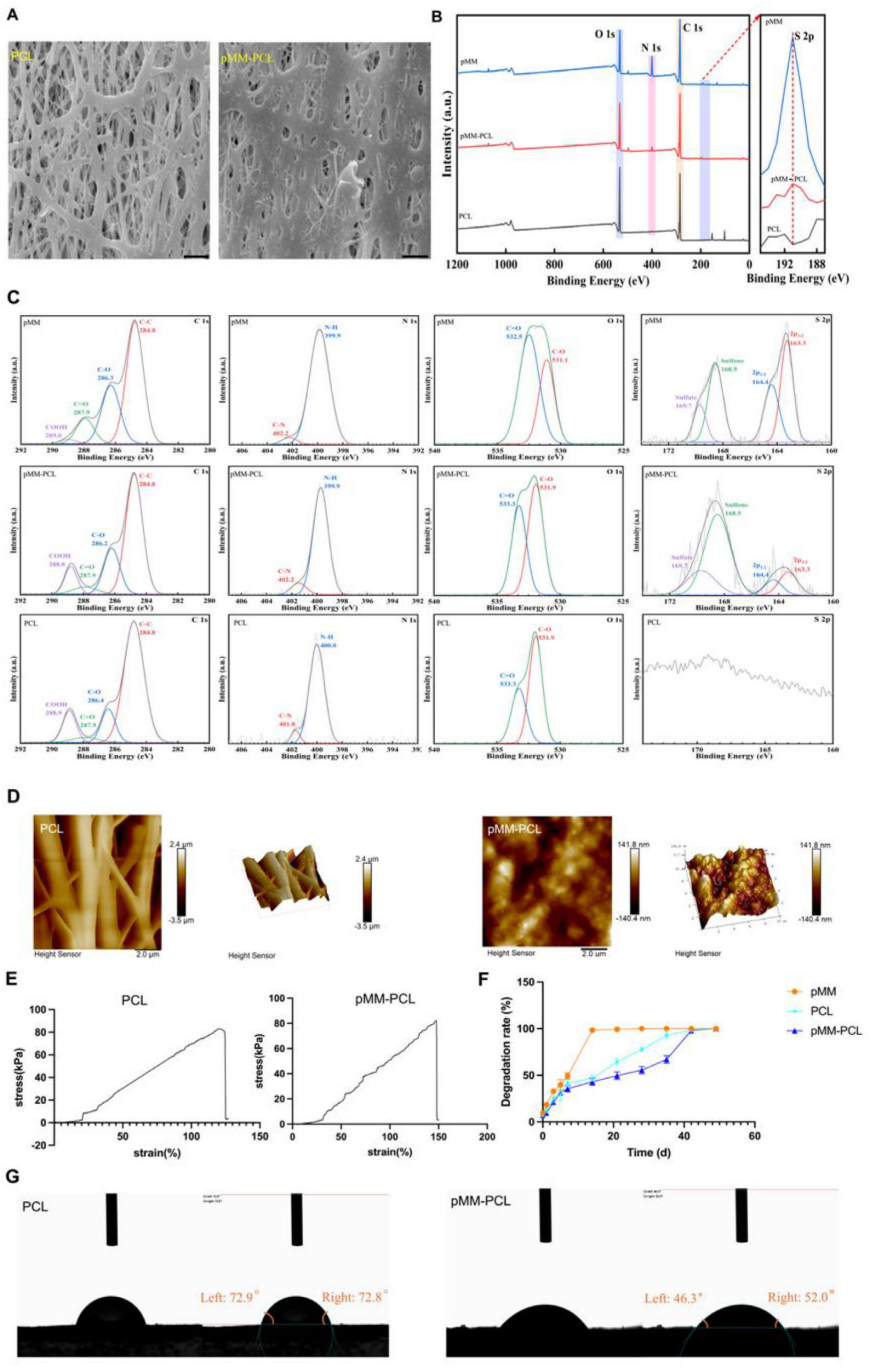
Characterization of pMM‐PCL A) Scanning electron microscope (SEM) comparison of PCL and pMM‐PCL. Scale bar: 500 nm (× 10). B,C) X‐ray Photoelectron Spectroscopy (XPS) analysis of PCL, pMM, and pMM‐PCL. D) Atomic Force Microscope (AFM) of PCL and pMM‐PCL. Scale bar: 2.0 µm (×50,00). E) Difference in tension test between PCL and pMM‐PCL (n = 3). F) Degradation rates of pMM, PCL, pMM‐PCL (n = 3). G) Hydrophilicity test of PCL and pMM‐PCL.

After the pMM and electrospinning with a suitable morphology were obtained, the both were further combined to fabricate the pMM‐PCL composite. Based on the summarized methods, ultrasonic co‐incubation was decided upon.^[^ ^34^ ^]^ Subsequently, we began to verify the success of its synthesis. First, as is revealed by SEM in **Figure** **4**A, pMM‐PCL and PCL appeared the obvious difference; the overall appearance of pMM‐PCL was less shiny due to the cell membrane. Second, the elemental composition of the composite material was detected. As is shown in Figure 4B‐C, pMM‐PCL includes Carbon, Oxygen, and Nitrogen and the “Sulfur” element, of which the “Sulfur” comes from pMM and is lacked in PCL, indicating that the composite material possesses both cell membrane and electrospinning. Third, Atomic Force Microscope (AFM) was performed aimed at confirming a complete combination of pMM and PCL from the outside in far from the simply surface binding (Figure 4D). The results presented the relatively flat surface with a significant reduction of surface drop in pMM‐PCL, compared to that of PCL. Moreover, it was hard for us to discern the original filamentous structure of PCL following the wrap of pMM. Hence, the AFM results showed that pMM not only coated every thread in PCL electrospinning but also filled most of the pores, which laid a solid foundation for the efficient adsorption of myelin debris by our biomaterial. In conclusion, the above results demonstrate the successful synthesization of an ideal pMM‐PCL composite bio‐scaffold.

To examine the applicability of the pMM‐PCL in SCI, validation on elastic modulus, degradation rate, and hydrophilicity were performed. Elastic modulus matching the spinal cord can avoid unnecessary damage caused by the contraction of the injured sites, appropriate degradation time allows the material to play its full role, and excellent hydrophilicity improves the overall biocompatibility of materials and their adhesion to spinal cord. Based on the stress‐strain results (Figure 4E), PCL increased the maximum stress remarkably to maintain full elastic deformation after coated by pMM, from 125 to 150Mpa, indicating a significant enhancement in the ductility of PCL. Since myelin debris remains for a long time after SCI, the degradation velocity of the composite material should be suitable.^[^ ^1^ ^]^ As is depicted in Figure 4F, pMM underwent complete degradation (99.35% ± 0.56%) within 13 d. However, with the incorporation of PCL (98.50% ± 1.13% completely degraded in 41 d), the degradation profile of pMM‐PCL was markedly different. The overall degradation time of pMM‐PCL (99.50% ± 0.44%) extended to 44 d, a substantial increase compared to pMM. As is presented in Figure 4G, the water contact angle of pMM‐PCL is conspicuously smaller than that of PCL (the water contact angle of pMM‐PCl is 46.3° on the left and 52.0° on the right and that of PCL is 72.9° on the left and 72.8° on the right). This decrease in the water contact angle clearly demonstrates that pMM remarkably improves the biocompatibility of PCL, which endows pMM‐PCL with greater potential for applications in the biomedical field. Moreover, adhesion force test was performed to demonstrate the adhesion of pMM‐PCL to spinal cord tissue (detailed data and discussion was listed in Supplementary Material S7, Supporting Information). Taken together, our composite material not only immobilizes the cell membrane but also has a longer action time, excellent ductility, and robust adhesion to the spinal cord, which meets the requirements for adsorbing myelin debris in a lasting way.

### 3.4 In Vitro Cell Experiments

Following synthesis of the biomaterials, the neuronal toxicity of the composites was first investigated, which directly determines their application in SCI. As is shown in Supplementary Material S1 (Supporting Information), compared with the Sham group (96.67% ± 4.09%), the activity of neurons was not affected after they were co‐cultured with pMM‐PCL (97.27% ± 2.25%). In view of the wide application of PCL in nerve regeneration, the data shows that pMM‐PCL can be applied in our study without compromising the biocompatibility of PCL.

#### 3.4.1 pMM‐PCL Mitigated the Inflammation by Adsorbing Myelin Debris and Regulating Macrophages

**Figure 5:**
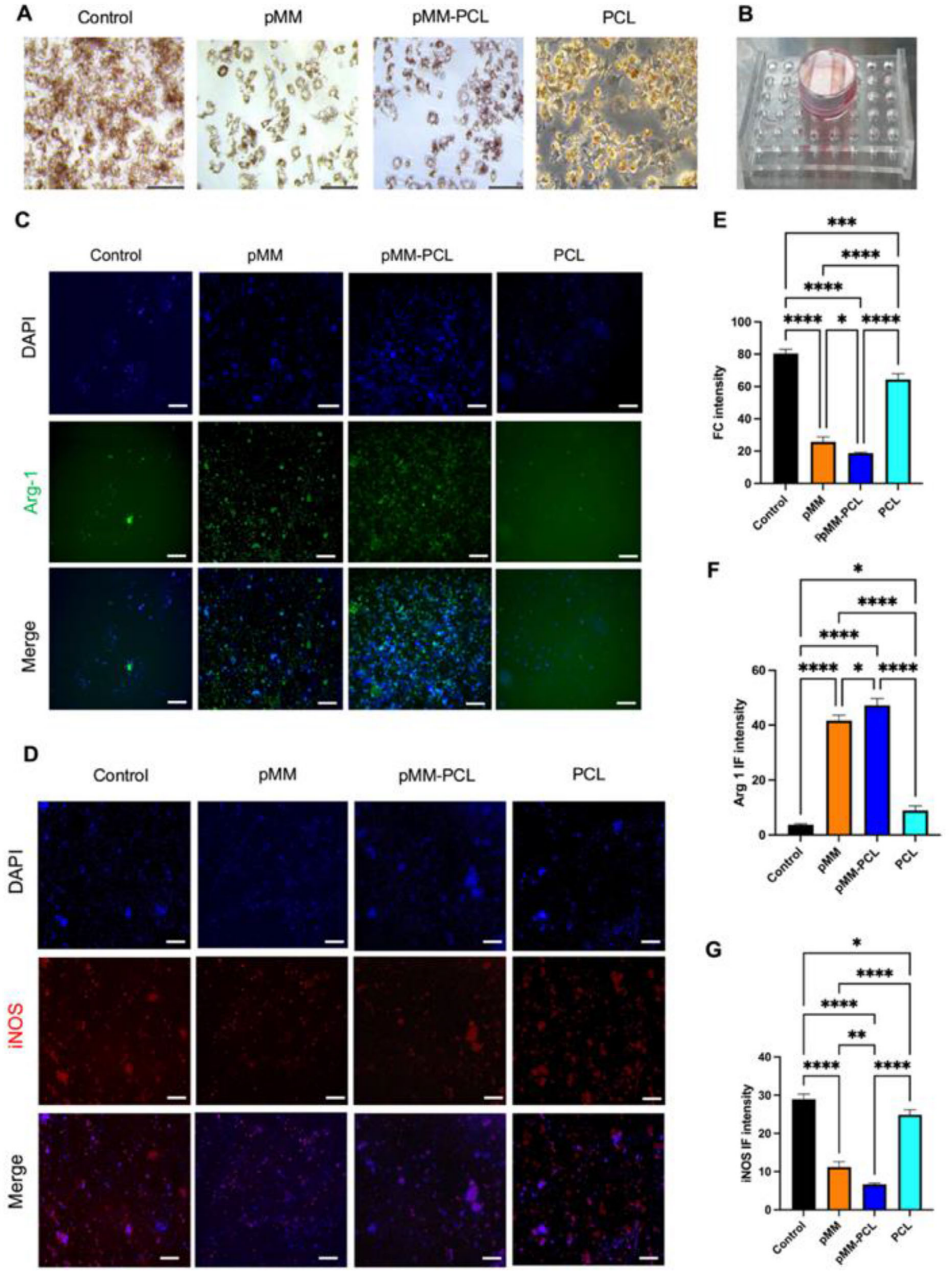
pMM‐PCL decreases the number of foam cells and regulates its proinflammatory effect. A) pMM‐PCL limits the formation of foam cells by adsorbing myelin debris. Scale bar: 200 µm (× 5). B) Schematic photo of pMM‐PCL's adsorption to myelin debris. C,D) pMM‐PCL improves the proinflammatory effect of macrophages. Scale bar: 100 µm (× 5). E–G) Qualification of foam cells, Arg1 and iNOS intensity (n = 3), assessed via one‐way ANOVA. *P < 0.05; **P < 0.01; ***P < 0.001; ****P < 0.0001.

Since pMM‐PCL was established feasible in SCI recovery, in vitro experiments of myelin debris were then carried out. To facilitate observation, the density of ORO staining was used to indirectly reflect the adsorption of myelin debris in the Control, PCL, pMM, and pMM‐PCL groups. Moreover, homogenate of spinal cord at 3‐dpi was picked to mimic the physiological microenvironment after SCI. As is depicted in **Figure** **5**A, PCL exhibited almost no adsorption on myelin debris and presented a high ORO staining density (64.33 ± 3.51), similar to that of the Control group (80.33 ± 2.51). However, the density of foam cells in the pMM group (25.66 ± 3.05) and the pMM‐PCL group (22.66 ± 3.51) was significantly decreased, indicating that pMM‐PCL retained the ability to adsorb myelin debris from pMM. After SCI, the acute and chronic inflammation induced by myelin debris is acknowledged as a crucial obstacle to nerve regeneration. Therefore, decreasing the amount of myelin debris could potentially mitigate the severe inflammation. The semi‐quantitative analysis of pro‐inflammatory and anti‐inflammatory markers of macrophages in the microenvironment showed that (Figure 5C‐D,F‐G), the IF intensity of Arg1 in the Control, pMM, pMM‐PCL and PCL groups were 3.77 ± 0.39, 42.30 ± 2.28, 44.84 ± 2.99, and 5.17 ± 0.28, respectively; the IF intensity of iNOS in the Control, pMM, pMM‐PCL and PCL groups were 28.96 ± 1.36, 11.15 ± 1.43, 6.64 ± 0.32, and 29.65 ± 0.69, respectively, reflecting a reduced level of inflammation in pMM‐PCL group owing to the robust adsorption of myelin debris.

**Figure 6:**
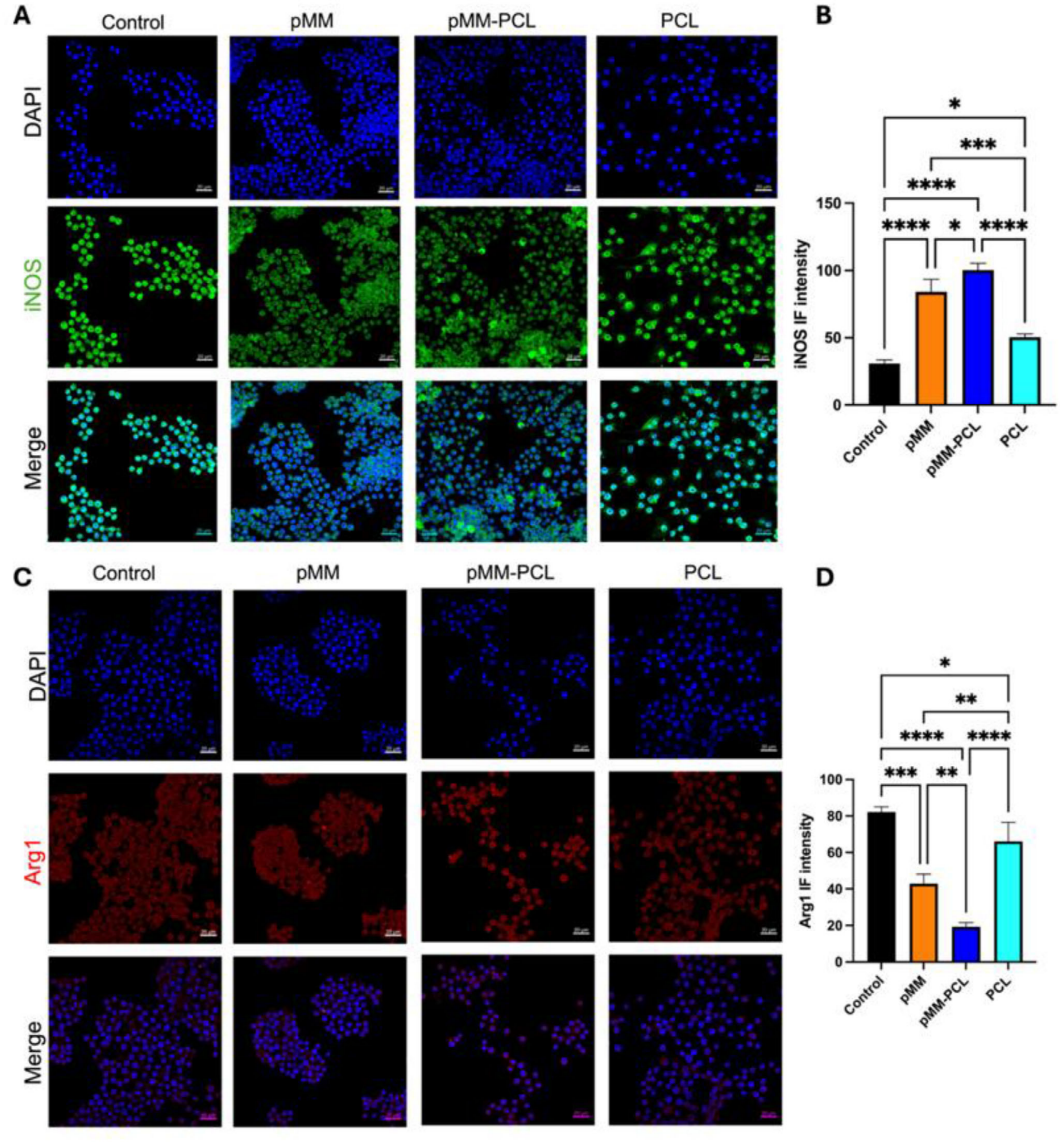
Myelin debris adsorbed on pMM‐PCL attracts macrophages and skews them to be proinflammatory. A) Dense pro‐inflammatory marker on pMM‐PCL after adsorbed myelin debris. Scale bar: 100 µm (× 5). B) Qualification of iNOS Intensity (n = 3), assessed via one‐way ANOVA. C) Sparse anti‐inflammatory marker on pMM‐PCL after adsorbed myelin debris. Scale bar: 100 µm (× 5). D) Qualification of Airg1 intensity (n = 3), assessed via one‐way ANOVA. *P < 0.05; **P < 0.01; ***P < 0.001; ****P < 0.0001.

In contrast, pMM‐PCL group, in **Figure** **6**A, showed the most serious inflammation on pMM‐PCL, since the macrophages were attracted by adsorbed myelin debris (the IF intensity of iNOS in the Control, pMM, pMM‐PCL, and PCL groups was 30.38 ± 2.63, 84.13 ± 9.31, 96.98 ± 2.22, and 50.44 ± 2.31, respectively). Due to the inability of plain slides to adsorb myelin debris, macrophages on them in the Control group demonstrated the highest expression of M2 polarization markers. Similarly, M2 polarization markers in the PCL group demonstrated low expression for inability to adsorb myelin debris (IF intensity of Arg1 in the Control, pMM, pMM‐PCL, and PCL groups were 82.15 ± 2.86, 42.99 ± 5.12, 19.25 ± 2.29, and 66.10 ± 10.39, respectively). These results demonstrated that our composite materials could regulate the overall inflammatory microenvironment, which was, at least, achieved by reducing myelin debris and foam cells. Based on in vitro experimental evidence, we hypothesize that when applied to spinal cord injury, the pMM‐PCL would exert therapeutic effects by adsorbing neurotoxic myelin debris and reducing foam cells, thereby establishing an anti‐inflammatory immune microenvironment at the injury site that facilitates the recovery of SCI.

**Figure 7:**
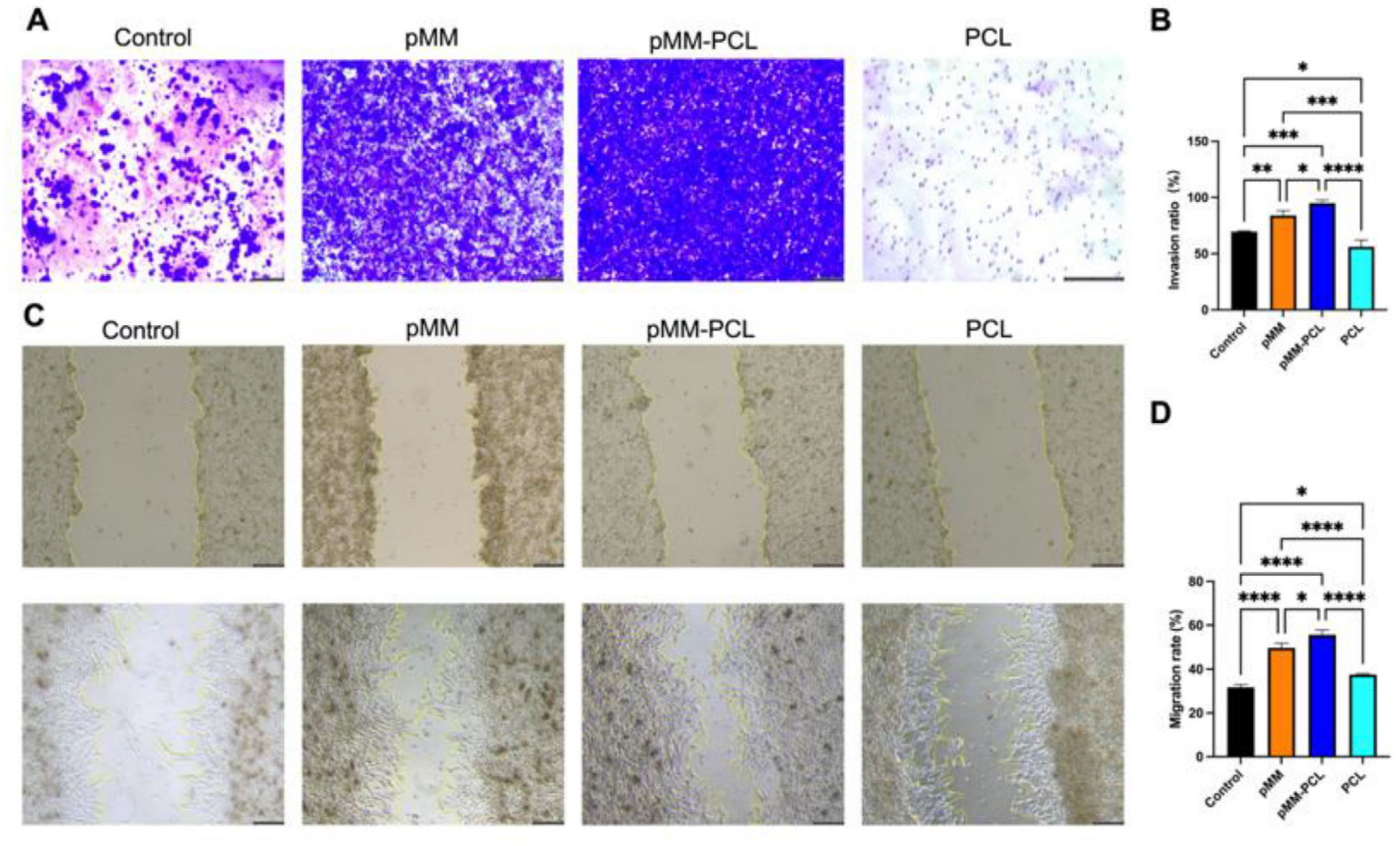
Myelin debris’ chemotaxis of macrophages and pMM‐PCL improves the direct inhibition of myelin debris on neurons. A) The invasion of macrophages in the Transwell assay. Scale bar: 100 µm (× 5). B) Quantification of migrating macrophages in the lower chamber (n = 3), assessed via one‐way ANOVA. C) Proliferation of PC12 co‐cultured with myelin debris in each group. Scale bar: 100 µm (× 5). D) Quantification of migrating PC12 in the wound healing assay (n = 3), assessed via one‐way ANOVA. *P < 0.05; **P < 0.01; ***P < 0.001; ****P < 0.0001.

Considering the severe inflammation on pMM‐PCL when co‐cultured with spinal cord homogenate, we postulated that the adsorbed myelin debris had the potential to induce macrophages to migrate toward pMM‐PCL. Therefore, Transwell assay was performed in vitro to validate this hypothesis. As is depicted in **Figure** **7**A‐B, the number of macrophages in the lower chamber was the highest in the pMM‐PCL group (96.59 ± 0.10), followed by a slightly smaller number in the pMM group (87.18 ± 1.85). In contrast, the macrophages count in the other two groups was significantly lower (the intensity in the Control and PCL groups were 69.62 ± 0.38, and 58.47 ± 0.06, respectively). These findings indicated that pMM‐PCL had the capability of attracting macrophages to migrate after adsorbing myelin debris. Therefore, it is reasonable to speculate that pMM‐PCL is likely to alleviate foam cells in the injured spinal cord by adsorbing myelin debris and attracting macrophages.

### 3.5 pMM‐PCL Alleviates Myelin Debris‐Mediated Direct Inhibition of Axonal Regeneration

As is mentioned above, myelin debris could impose a substantial inhibition on axonal growth cones, which is detrimental to the recovery of SCI. To verify the intervene of pMM‐PCL on this inhibitory effect, the proliferation of PC12 (simulating neurons) in the microenvironment filled with myelin debris across different groups was explored. Figure 7C‐D indicates that the proliferation of PC12 in the pMM‐PCL group, as well as in the pMM group, was much better than that in the control group and the PCL group (the migration ratios of the Control, pMM, pMM‐PCL, and PCL groups were 31.68% ± 1.26%, 52.30% ± 0.36%, 52.96% ± 0.67%, and 37.49% ± 0.50%, respectively), showing strong scratch healing. The experimental data demonstrate that pMM‐PCL significantly alleviates myelin debris‐mediated inhibition of axonal regeneration, suggesting its potential therapeutic value in SCI.

### 3.6 In Vivo Experiments

#### 3.6.1 pMM‐PCL Reduced the Number of Foam Cells in the Center of Grey Matter

**Figure 8:**
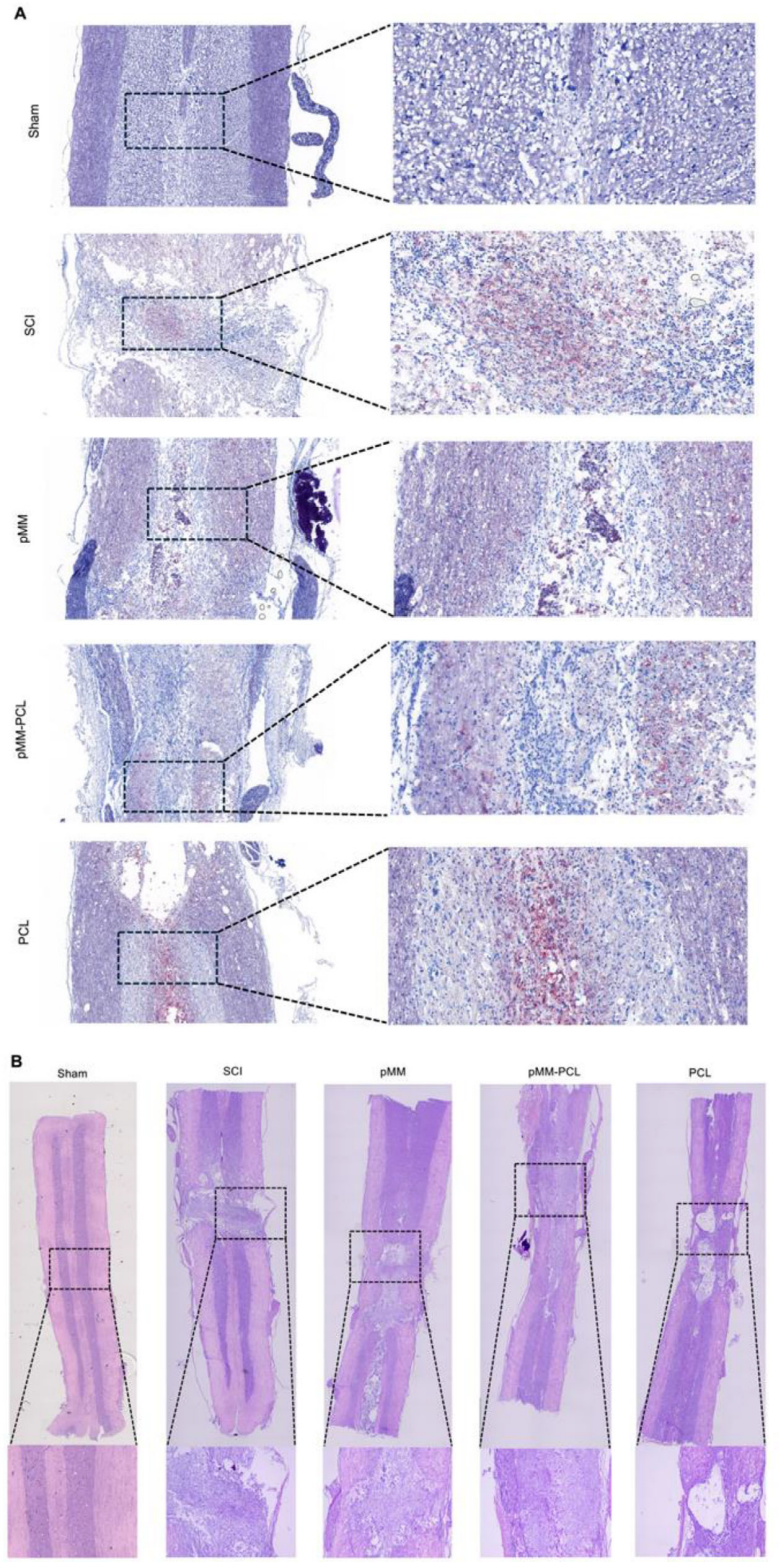
pMM‐PCL results in foam cells moving toward the spinal cord edge and promotes SCI repair. A) ORO staining in each group. The figures on the left are at low magnification, Scale bar: 500 µm (× 5); the others on the right are at high, Scale bar: 500 µm (× 10). B) Masson staining. The upper are low magnification figures. Scale bar: 500 µm (× 5), and the below are enlarged ones. Scale bar: 500 µm (× 10).

The HE histopathological results of major organs (list in Supplementry Material S1) showed good biosafty of pMM‐PCL, suggesting its potential in SCI treatment. In verifying the effect of pMM‐PCL on the foam cells in vivo, ORO staining was performed on frozen sections of the spinal cord at 6‐weeks post injury (6‐wpi). In light of previous studies, foam cells appeared at the edge of the lesion from the 7‐dpi, gradually converged toward the lesion core. and eventually accumulated in the injury core forever, which was so‐called “centrality”.^[^ ^35^, ^36^ ^]^ Therefore, as is shown in **Figure** **8**A, ORO staining in SCI group at 6‐wpi was predominately concentrated in the lesion core at a high density due to the presence of abundant myelin debris. The situation was similar to that in the PCL group. These results reflected the inability of PCL to adsorb myelin debris in vivo. In the pMM‐PCL and pMM groups, however, ORO staining was scattered toward the spinal cord margin and remained a little in the lesion core, indicating that myelin debris as well as foam cells were effectively shifted outward by the composite material. Moreover, the migrating foam cells to the margin in the pMM group were not as many as those in the pMM‐PCL group, which may be attributed to the fact that cell membrane alone failed to fix on one point. All in all, these results showcased that the composite material applied in the injured spinal cord effectively adsorbs myelin debris, which ultimately leads to a reduction in the number of foam cells at their original site.

#### 3.6.2 pMM‐PCL Alleviated the Formation of Cavity and Scar

**Figure 9:**
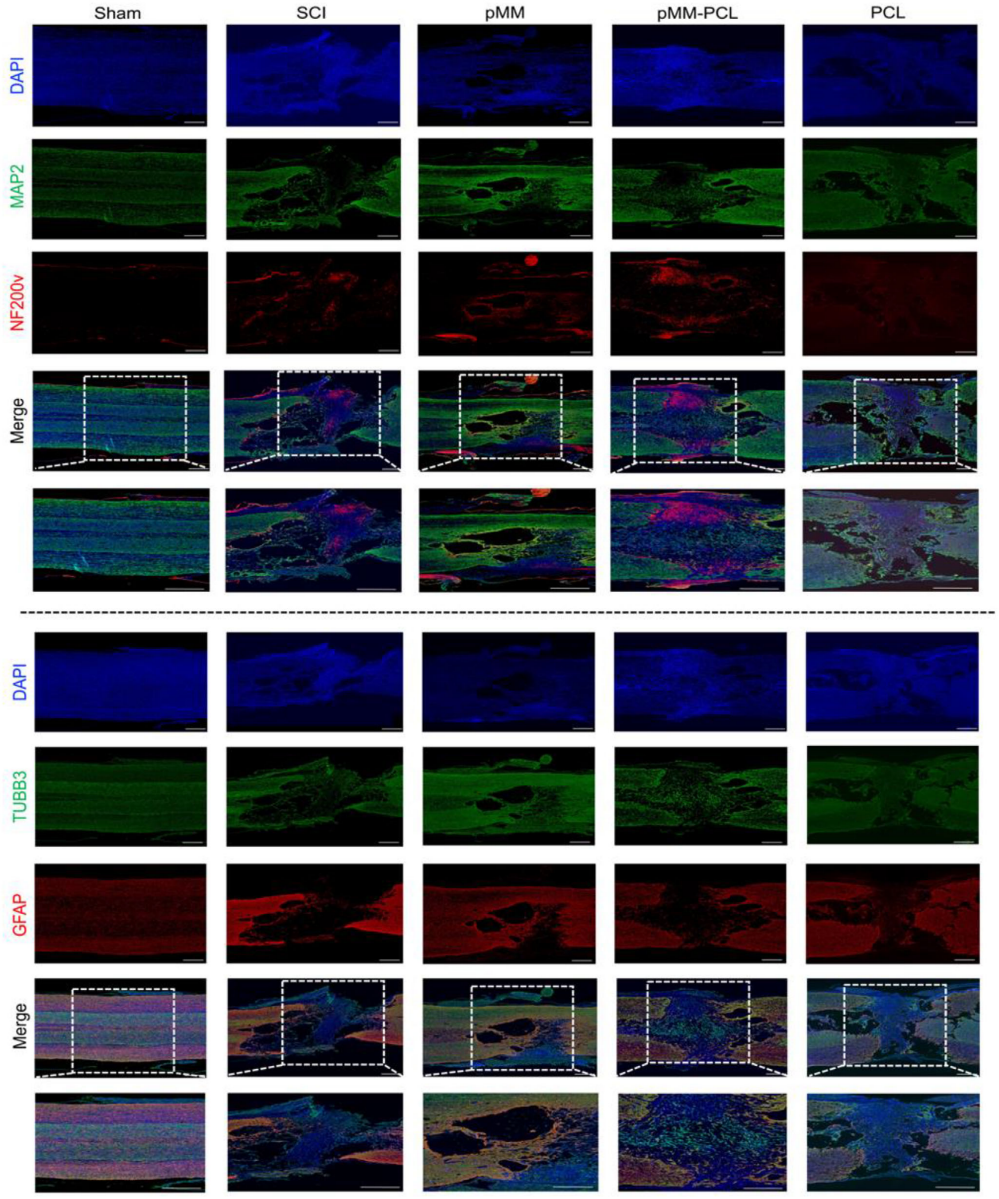
pMM‐PCL promotes neuronal regeneration and reduces scar formation in vivo. 6‐wpi, immunofluorescence analysis of DAPI (blue), MAP2 (green), NF200 (red), TUBB3(green), and GFAP (red) in four groups was performed. Low magnification image, Scale bar: 500 µm (× 4). Enlarged image, Scale bar: 500 µm (× 10).

**Figure 10:**
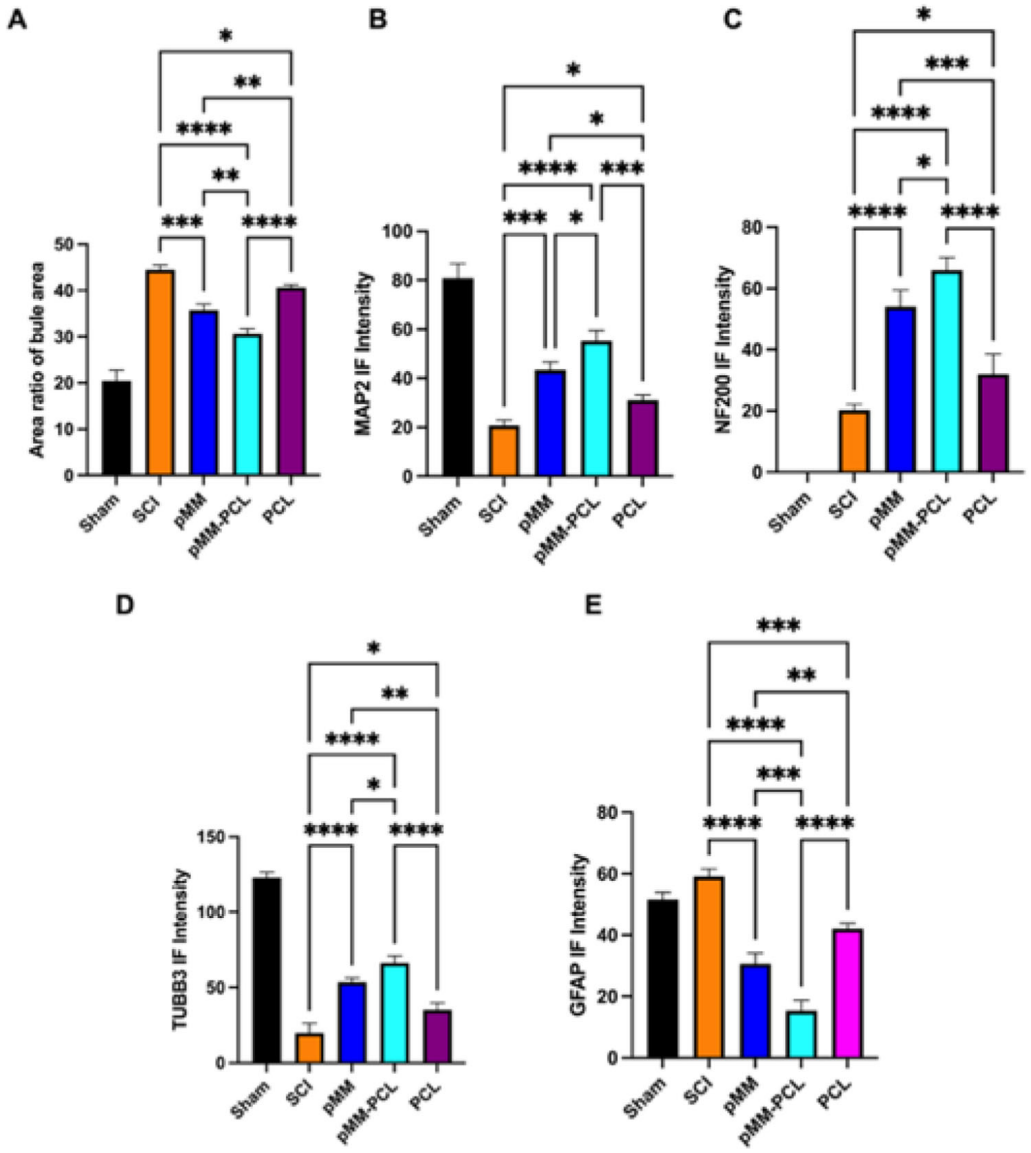
Benefits of pMM‐PCL on SCI recovery. A) Qualification of blue area in Masson staining (n = 3), assessed via one‐way ANOVA. B) Qualification of MAP2 immunofluorescence Intensity. C). Qualification if NF200 immunofluorescence Intensity (n = 3), assessed via one‐way ANOVA. D) Qualification of tubb3 immunofluorescence Intensity (n = 3), assessed via one‐way ANOVA. E) Qualification of GFAP immunofluorescence Intensity (n = 3), assessed via one‐way ANOVA. *P < 0.05; **P < 0.01; ***P < 0.001; ****P < 0.0001.

Numerous studies have shown that myelin debris or foam cells can further the scar formation after SCI, hinder the connection of nerve axons at both ends of the injury, and are not conducive to the recanalization of sensory/motor signals.^[^ ^2^, ^37^ ^]^ In the aspect of exploring the effects of pMM‐PCL on saccring, Masson staining of the spinal cord at 6‐wpi was performed, which could reflect the collagen scar formation. According to Figures 8B and 10A, the rats in the SCI group presented an unsatisfying repair trend at 6‐wpi, showing obvious tissue cavities and scar (reflected by blue areas intensity, 44.49 ± 0.98). However, the rats in the pMM‐PCL group exhibited the most satisfying recovery of tissue among all groups, with significant improvements in cavity, rare disconnection at both ends of lesion, and improved scar formation (30.65 ± 1.06). Combined with the validation of pathology, GFAP fluorescence was also used to identify the effect of pMM‐PCL on glial scar. As is shown in **Figure** **9**, in line with the pathological results, the fluorescence intensity of GFAP (green) in the pMM‐PCL group showed the lowest (15.27 ± 3.51) among all groups with much regenerated tissue bridging the lesion (reflected by bule nucleus after DAPI staining). And in the pMM group, cavity and scarring were improved (30.65 ± 3.38), though not as significantly as the pMM‐PCL group. Therefore, the results shown above suggest that pMM‐PCL ameliorates tissue discontinuity and scar formation during SCI repair, which is achieved by reducing myelin debris or foam cells.

#### 3.6.3 pMM‐PCL Promoted Neuronal Growth and Motor Function Recovery

**Figure 11:**
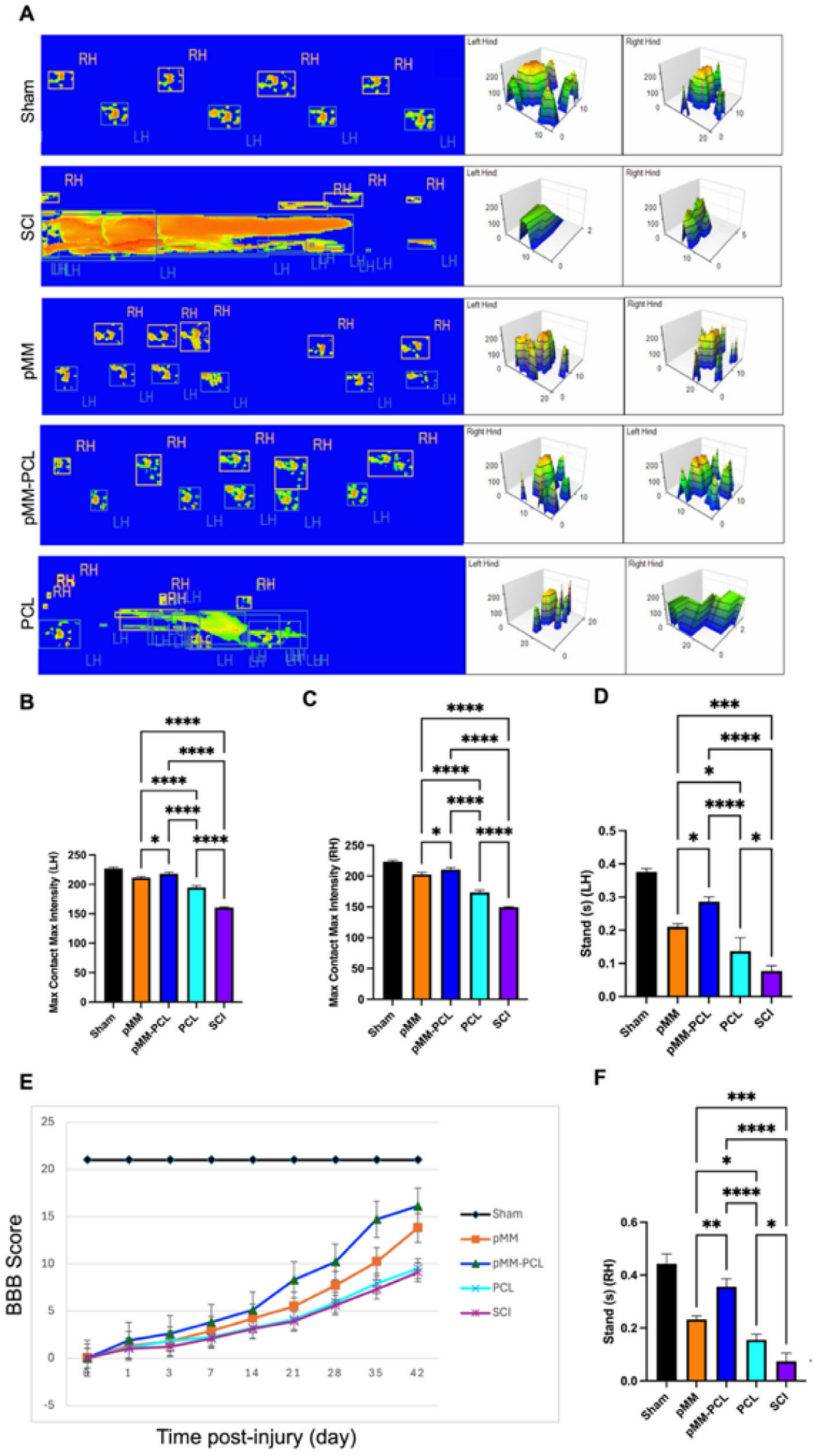
pMM‐PCL promotes functional recovery in rats with SCI. A) Gait analysis of both hind limbs used to reflect functional recovery. B,C) Quantification of ground area of both hind limbs during a walking cycle (n = 3), assessed via one‐way ANOVA. D,E) Quantification of grounding time of both hind limbs during one walking cycle (n = 3), assessed via one‐way ANOVA. F) BBB score used to reflect the recovery of motor function in both hind limbs (n = 3), assessed via one‐way ANOVA. *P < 0.05; **P < 0.01; ***P < 0.001; ****P < 0.0001.

The regeneration of neurons plays a crucial role in the process of SCI repair, which is directly related to the recovery of sensory and motor functions.^[^ ^38^ ^]^ Therefore, in verifying the effects of pMM‐PCL on neuronal regeneration after SCI, MAP2, and TUBB3 for labeling newborn neurons and NF200 for marking newborn axons were performed at 6‐wpi. As is shown in Figures 9 and **10**B‐D, the pMM‐PCL group showed significantly stronger neuronal regeneration than other groups (the MAP2 IF intensity in the SCI, pMM, pMM‐PCL and PCL groups were 20.81 ± 2.02, 43.47 ± 3.15, 55.32 ± 4.19, and 31.11 ± 2.07 respectively; the TUBB3 IF intensity in the SCI, pMM, pMM‐PCL and PCL groups were 19.84 ± 6.32, 53.41 ± 3.07, 66.13 ± 4.68, and 35.01 ± 4.83, respectively; the NF200 IF intensity in the SCI, pMM, pMM‐PCL and PCL groups were 20.23 ± 2.02, 54.05 ± 5.42, 65.93 ± 4.13, and 29.03 ± 1.92, respectively), the three antibodies occupying the lesion well. In contrast, the PCL group and the SCI group showed poor neuronal regeneration, with little shadow of newborn neurons and axons. However, excellent regeneration or repair of neurons may not equal ideal functional recovery. Therefore, further assessment of motor function was carried out. According to gait test for both hind limbs (**Figure** **11**A‐E), only SCI rats in the pMM‐PCL group had the best motor function recovery at 42‐dpi, with regular double hind leg footprints, long standing time (Right: 0.37 ± 0.02s; Left: 0.28 ± 0.01s) and large ground contact intensity (Right: 210.69 ± 3.41; Left: 217.90 ± 2.66), despite a gap compared to the Sham group (standing time: 0.44 ± 0.03 of the right hind legs and 0.37 ± 0.01 of the left hind legs; ground contact intensity: 223.57 ± 2.15 of the right hind legs and 226.89 ± 2.34 of left hind legs). Comparatively speaking, the rats in SCI and PCL groups showed disorderly footprints with the tail in tow, whose analysis of walking behavior details were also indicative of poor functional recovery. The results of functional recovery could also be manifested in BBB score (Figure 11F). Among all groups, rats with SCI were 0 at 0‐dpi, with rats' paralysis of both hind limbs, indicating that the SCI contusion model was successfully established. At 14‐dpi, only the rats in the pMM‐PCL group could reach a score of 5 among all groups. Similarly, the score of the pMM‐PCL group was the highest (16.04 ± 0.09) at 42‐dpi, indicating that pMM‐PCL could effectively facilitate functional recovery after SCI. In conclusion, pMM‐PCL could promote the recovery of motor function in rats with SCI by enhancing neuron regeneration.

#### 3.6.4 pMM‐PCL Rewrites Multiple Metabolic Processes in SCI

All the above results show the obvious influence of pMM‐PCL on SCI at macroscopic level such as pathology, but it is still unknown which molecules or pathways drive these changes. Therefore, lipomic and proteomic analysis were used on 6‐wpi.

**Figure 12:**
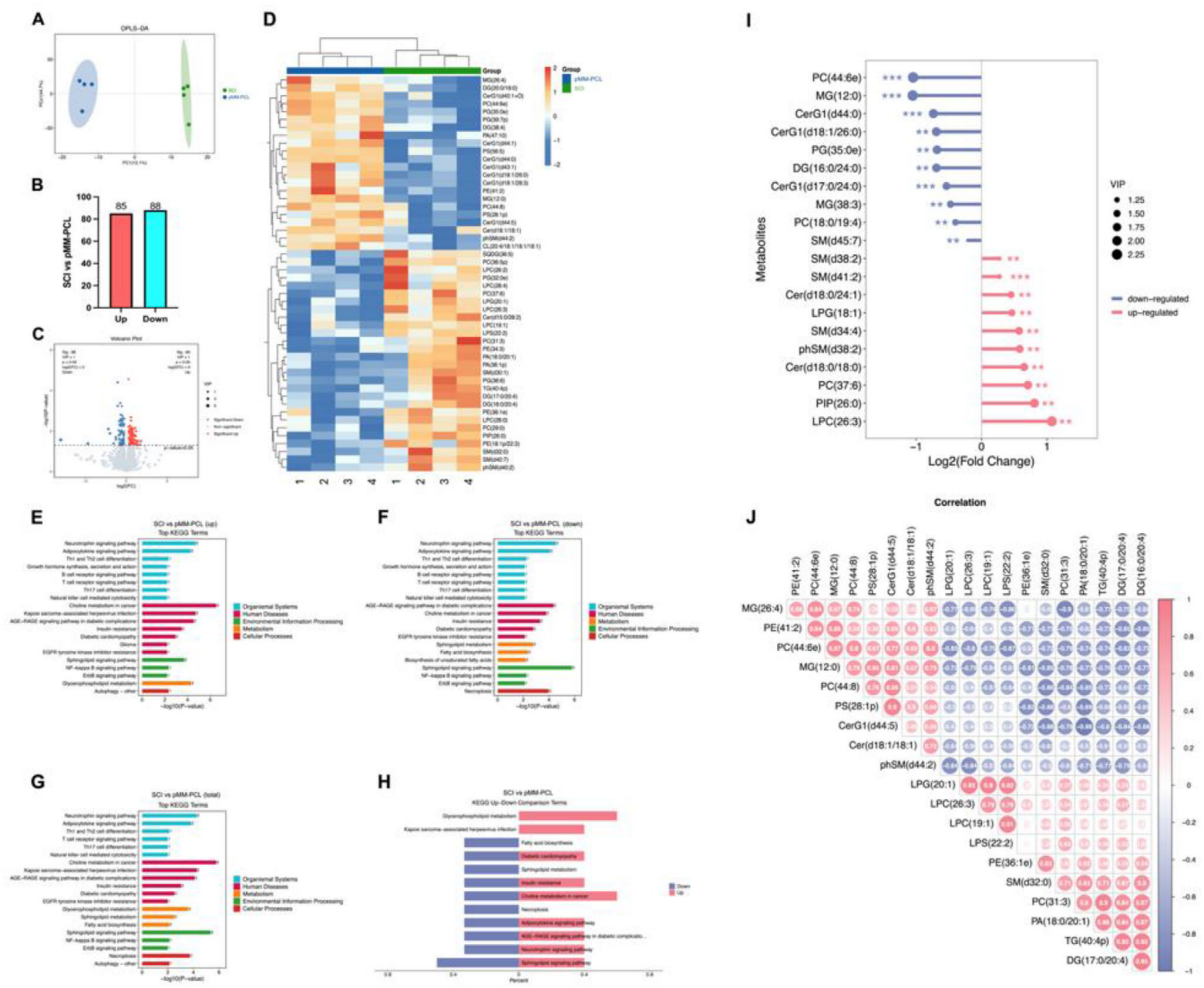
pMM‐PCL changes original lipid metabolism in SCI. A) PCA analysis of lipidomics data from 8 samples. B) Statistics of differential screening results. C) Volcano plot shows differentially expressed metabolites. D) Heatmap shows differences in lipid composition between samples. E‐H): KEGG pathway enrichment analysis. I) The lollipopmap visually shows differential metabolites and their foldchange values. J) Correlation between differential metabolites.

**Figure** **12**A‐C showed that the lipid components inside the spinal cord at 6‐wpi were altered after the application of pMM‐PCL. After determining that these changes were statistically significant, we counted 173 changed lipid components in total, among which 85 were down‐regulated and 88 were up‐regulated. To more intuitively show the differences of lipid metabolites before and after the pMM‐PCL application, hierarchical clustering was performed with the expression levels of the top few significantly different metabolites with the smallest p‐value. In this study, lipids like TG, LPC, and SM, which were proved up‐regulated after SCI, decreased after the intervention of pMM‐PCL, and lipids like CerG, which had been proved down‐regulated, were reduced after the application of pMM‐PCL (Figure 12D, I).^[^ ^39^ ^]^ Figure 12E‐H showed pathway enrichment analysis of differential metabolites between the SCI group and the pMM‐PCL group, which contributes to a deeper understanding of the influence behind the changes in the two groups. It was evident that the pathway related to fatty acid biosynthesis was significantly down‐regulated in the SCI group compared with the pMM‐PCL group, which could be attributed to the phagocytosis of myelin debris by macrophages. After SCI, myelin debris, rich in cholesterol, phospholipids, and glycolipids, is engulfed and digested into large amounts of free fatty acids, which will inhibit fatty acid self‐biosynthesis in a negative feedback way.^[^ ^40^ ^]^ Besides, Kyoto Encyclopedia of Genes and Genomes (KEGG) analysis showed that pathways associated with neurotrophins, and inflammation (reflected by NF‐κB) were obviously changed due to pMM‐PCL, which was parallel to the results of in vitro research above. Figure 12J showed the correlation of some lipid components that differed significantly between two groups. Taken together, the lipidomic analysis effectively demonstrated our successful adsorption of myelin debris in SCI.

**Figure 13:**
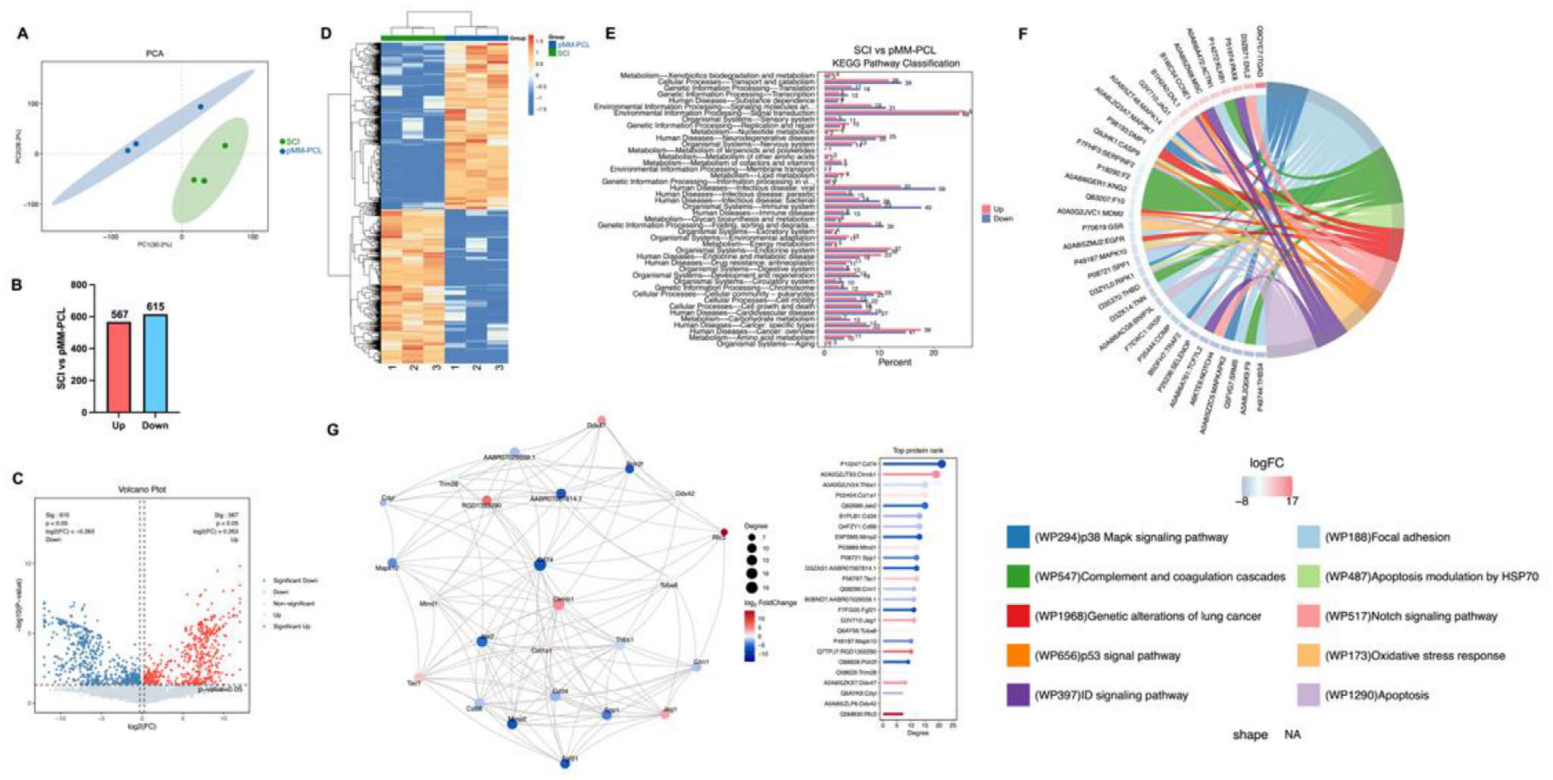
pMM‐PCL changes the protein metabolism in SCI. A) PCA analysis of proteomics data from 6 samples. B) Statistics of differential screening results. C) Volcano plot shows differentially expressed metabolites. D) Heatmap shows differences in protein expression between samples. E) KEGG pathway enrichment analysis. F) Wikipathways enrichment analysis reinforces and supplements KEGG analysis. G) Differential protein interaction network shows the relationship between the proteins.

Similar to the results of lipidomic, as shown in **Figure** **13**A‐D, the application of pMM‐PCL resulted in significant changes in protein composition. After determining that these changes were statistically significant, we counted a total of 1172 altered proteins, of which 615 were up‐regulated and 567 were down‐regulated after pMM‐PCL application. Then KEGG analysis and Wiki Pathways analysis demonstrated that these altered proteins might be involved in multiple aspects such as metabolism, cellular processes, and human diseases. As is shown in Figure 13E‐F, the SCI group had more proteins related to lipid metabolism, neurodegenerative diseases, and inflammation than the pMM‐PCL group, indicating less myelin debris in the pMM‐PCL group. In order to better understand the crosstalk among these changed proteins, the top 25 proteins with high connectivity were selected to draw a network diagram. As is shown in Figure 13G, the top three distinguishing proteins with high connectivity are CD74, Ctnnb1, and Thbs1. After literature retrieval, the three were found to be associated with lipid hemostasis, macrophage migration, glial and fibrous scar formation, and nerve injury.^[^ ^41^, ^42^, ^43^, ^44^, ^45^, ^46^ ^]^ As for macrophage migration, the results of protein metabolism analysis could help explain the long‐time staying of macrophage in the lesion core after it engulfs myelin debris. Consequently, the result of proteomics indirectly reflected the situation of lipid metabolism and its influence on SCI pathology after pMM‐PCL application, which is in accordance with experimental results in our study and previous studies.^[^ ^3^ ^]^

**Figure 14:**
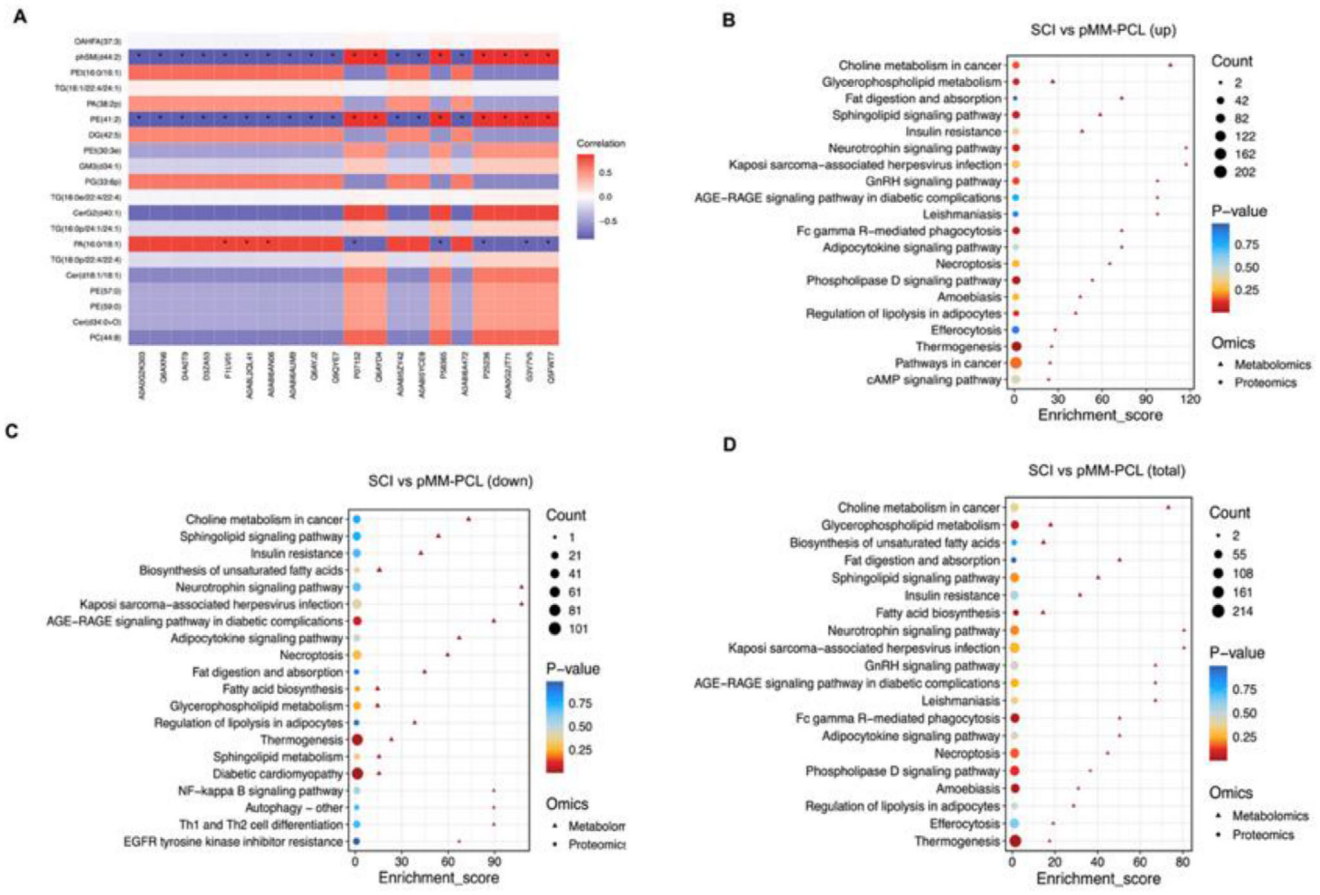
Conjoint analysis of lipidomics and proteomics. A) Correlation analysis of differential protein components and differential lipid components. B‐D) Analysis of common pathways for differential proteins and differential metabolites.

Given the intricate interactions between lipid metabolism and protein metabolism in biochemistry, a joint analysis of both omics results above was conducted. As is depicted in **Figure** **14**A, PE, PG, DG, CerG, and PA et al. were the top lipids closely related to protein metabolism, consistent with the results obtained above. Figure 14B‐D illustrated pathways influenced jointly by differently expressed lipids and proteins. It was obvious that most of these pathways were linked to lipid metabolism, involving lipid catabolism and fatty acid biosynthesis et al. Notably, the Fc γ R receptor, involved in myelin debris phagocytosis, exhibited decreased expression following pMM‐PCL application, indicating the reduced load of myelin debris in the injured spinal cord with the help of pMM‐PCL. Therefore, it can be concluded that pMM‐PCL could effectively remove myelin debris in SCI and regulate lipid metabolism caused by it.

## 4 Discussion

Aimed at myelin debris and foam cells, the present study has innovatively proposed the concept of “myelin debris adsorption”, through which the negative effect from myelin debris and foam cells could be attenuated simultaneously. Based on this concept, we have designed the biomaterial composed of macrophage membrane for adsorbing myelin debris and PCL electrospinning as a holder, which directly targets the pathology of SCI and provides a promising new strategy for SCI recovery.

The application mode and purpose of cell membrane in SCI were fully extended in this study. Macrophage membranes were applied outside the injured spinal cord to adsorb myelin debris. To improve their phagocytic efficiency, macrophages were pretreated with myelin debris to acquire more relevant receptors. The experimental results showed that the composite biomaterial composed of appropriate PCL electrospinning and pretreated macrophage membranes successfully achieved myelin debris adsorption. In vitro, pMM‐PCL reduced foam cells, alleviated inflammation, and mitigated nerve inhibition through adsorbing myelin debris. In vivo, foam cells disperse toward spinal cord margin in the pMM‐PCL group for myelin debris’ adsorption, consistent with the smallest blue area in Masson staining and promising functional regeneration. The shifted position of the foam cells from the lesion core to margin alleviates the serious microenvironment in the grey matter, endowing the repair of the grey matter a priority. Frontier studies have shown that injured corticospinal tracts in the white matter indeed rarely regenerate or achieve functional recovery after SCI. Conversely, it is the propriospinal neurons in the center of the spinal cord that are of regenerative significance.^[^ ^47^ ^]^ The propriospinal neurons are intrinsic neurons located within the grey matter center of the spinal cord. They are responsible for the transmission of neural signals within the spinal cord and possess remarkable plasticity, playing the pivotal role of the functional recovery after SCI.^[^ ^48^, ^49^ ^]^ Therefore, although myelin debris and foam cells migrating to the surface of the spinal cord may be unfavorable to the repair of injured white matter in the study, it was conductive to the restoration of the grey matter or propriospinal neurons.

In this experiment, the topological electrospinning scaffold made of PCL was used as the carrier of the cell membrane, and its parameters were strictly selected. In the lead‐up to the experiment, several kinds of common concentration and molecular weight were explored. Finally, mass fraction at 12% and molecular at 8w were selected after the comprehensive evaluation of the number of beads, diameter, and pore size. Based on the conditions above, the prepared electrospinning boasts a small number of beads, relatively large diameter and pores, long retention time on the surface of the spinal cord, and strong ability to regulate inflammation. Despite the widespread use of oriented structure, for its recognizable benefit to guide the growth of newborn axons, our team chose the topological electrospinning instead of the orientation one in this experiment.^[^ ^50^ ^]^ The reasons are as follows: First, the primary objective of this experiment is to remove the myelin debris that persists for a long time after SCI and to reduce foam cells in the lesion core. The topological electrospinning not only has an advantage of loading and anchoring more cell membrane for its intricate network structure, but also contributes to macrophages’ infiltration and polarization toward an anti‐inflammatory phenotype, which also escapes the possibly unnecessary inflammatory responses caused by the composite material. Second, as is mentioned before, propriospinal neurons in the center are critical in function recovery. Therefore, oriented electrospinning on the surface of the injured spinal cord may have little effect on the induction of such neurons. Instead, it is our composite material through reducing foam cells in the lesion core that can contribute to the regeneration of these neurons.

Combining the multi‐omics results and the results of in vitro and in vivo experiments, we could easily conclude that these omics changes were the mirror of the pathological process of SCI, including myelin debris phagocytosis, lipid overload, macrophage migration and the following inflammation, scar, and nerve injury in SCI. Furthermore, in addition to macrophages, these omics changes may also be involved in astrocytes.^[^ ^51^ ^]^ This was consistent with previous studies that myelin debris was involved in multi‐secondary injuries mediated by macrophages, microglia, astrocytes, oligodendrocytes, vascular endothelial cells and other cells after SCI.^[^ ^16^ ^]^ All in all, myelin debris plays a critical role in the pathology of SCI, which activates multiple cells involved in SCI and is the common source of secondary injuries in particular inflammation. Therefore, the “simple” biomaterial could be versatile. Besides, a question that the first “centrality” and the upcoming “immobility” of foam cells in SCI deserves further investigation. In the characteristics of “centrality” and “immobility”, foam cells appeared originally at the edge of the lesion from 7d and gradually migrated and accumulated in the core of lesion permanently, which was mainly induced by myelin debris.^[^ ^37^ ^]^ Thus, foam cells in the injured core are not in situ but migrate from the lesion margin, accounting for the phenomenon that a large number of macrophages loaded with myelin debris exist in the core of the grey matter after SCI rather than the white matter with much more myelin coating axons.^[^ ^52^ ^]^ Despite the fact that some potential molecules were reflected in the omics results, such as CD74, more molecules and pathways are still waiting to be explored.

It is evident that the number of mitochondria in foam cells (48.33 ± 1.15) in vitro is about threefold higher than that in macrophages (15.33 ± 2.08). Additionally, within these mitochondria, there are scarcely any visible cristae, and some mitochondria have even undergone fragmentation (Figure 2B). Parallelly, St‐Pierre et al. have found similarly that mitochondria in the macrophages near the inured spinal cord core exhibit a greater quantity and morphology without cristae.^[^ ^53^ ^]^ Recent study on mitochondrial metabolism indicates that mitochondria can be divided into two functionally distinct subpopulations through fusion and division, ensuring a balance between oxidative and reductive metabolism.^[^ ^54^ ^]^ One subset rich in ATP synthases has intact cristae and focuses on the oxidative phosphorylation to generate ATP, while the other one rich in P5CS (pyrroline‐5 carboxylate synthetases) lacks the cristae and tends to the reductive metabolism. Therefore, the relevant considerations are as follows. First, the alteration in mitochondrial number and morphology may be related to the elevated demand for the reduction reaction within foam cells because large amounts of oxidized lipids that are endocytosed by macrophages need to be deoxidated into neutral lipids for storage. Second, it is conspicuous that some mitochondria have been broken, and mitochondrial damage, ROS, and inflammation are closely linked, so it is likely that the negative effects of foam cells are related to severe mitochondrial dysfunction.^[^ ^55^ ^]^ Third, it has been shown that the level of lipid autophagy in myelin‐loaded macrophages declined at 3‐dpi, which partly accounts for the failed elimination of intercellular lipid of foam cells.^[^ ^8^ ^]^ Therefore, since mitochondria take part in the lipid autophagy within macrophages after SCI, there arises a contradiction between the macrophages possessing more mitochondria and its poor performance of lipid catabolism. In‐depth exploration is thus essential.

Despite the carefully performed experiment and satisfactory results, we still acknowledged the existence of some limitations acquiring further exploration. First, although the restoration of the grey matter was prioritized for the moment, the migrated myelin debris or foam cells were still expected to be removed from injured spinal cord thoroughly, leaving room for the recovery of white matter to achieve the complete SCI recovery. Hence, it remains a question how to improve the adsorption capacity of biomaterials for myelin debris, and where myelin debris or foam cells move subsequently. Aimed at the improvement of the adsorption capacity, chemical or physical methods could be given a priority, such as the attraction between positive and negative ions. And regarding the whereabouts of myelin debris or foam cells, it is desirable that the adsorbed myelin debris could be removed by a permeability gradient pump from the spinal cord or be reused as raw materials for the myelination of new neurons. Second, as an initial exploration of the novel concept, PCL electrospinning was chosen as the media to hold pMM, but the degradation rates of pMM‐PCL are not well synchronized. Thus, it is calling for the better vectors, like hydrogel and biological matrix material et al., which can also promote SCI repair muti‐dimensionally through loading various effectors.^[^ ^56^, ^57^, ^58^ ^]^ Third, omics analysis had better be cell specific. The differentially expressed molecular or pathways in this study were shared by several cells, which made itself a stumble for us to accurately identify the metabolism of specific cells, especially to distinguish microglia from macrophages. In addition, to demonstrate the long‐term effect of our biomaterial, we performed the omics analysis on 6‐wpi, leading to the lack of the data in the acute and chronic phase, which were also the key points for SCI. In the future, omics analysis in unique cells and rounded period should be performed.

## 5 Conclusion 

In this study, aimed at myelin debris and foam cells in SCI, we put up a novel concept of “myelin debris adsorption”. Based on this strategy, biomaterial composed of appropriate PCL electrospinning and pretreated macrophages membranes was designed by the present authors, which was established successfully cleaning myelin debris from injured spinal cord, reducing foam cells in the lesion core and finally promoting functional recovery of SD rats. Apart from pathological experiments, lipidomic and proteomic analysis were also performed, further reinforcing the validation. In summary, this study advances the research of myelin debris and foam cells in SCI and provides a new perspective for SCI recovery.

## Conflict of Interest

The authors declare no conflict of interest.

## Author Contributions

Y.C.Z. was responsible for the design of the experimental procedure, verified the composite material, participated in the whole process of experiments and wrote the article, and was the main contributor. T.X., together with Y.Y.Z., was responsible for the preparation and validation of cell membranes, P.C.L. electrospinning, and the design of animal experiments. Z.C.W. was responsible for photographing the in vitro cell experiments. Z.Z.Y. was responsible for data collection for the experiments. C.W.Y. was responsible for all data integration and collating. N.C. was responsible for the the language review and full‐text editing of manuscripts. X.Q.C. was responsible for reviewing the correctness of the experimental design, the rigor of the experimental verification, and the final review of the paper.

## Ethical Approval

All experimental procedures were performed in accordance with the Guide for the Care and Use of Laboratory Animals of the National Research Council. The animal ethics approval was approved by the Animal Care and Use Committee of Nantong University (Nantong, China; 220 203 322/22 020 214 810).

## Animal Welfare Protection

Y.C.Z., T.X., Z.C.W., and Z.Z.Y. are jointly responsible for ensuring the welfare of all animals in this study, ensuring that no experimental animals are treated unfairly. Throughout the experiment, the animals lived in an environment with sufficient food and water and were allowed to move regularly in a large area. Our team has over 12 years of experience in animal experiments and animal model establishment. The SD rats with spinal cord injury received meticulous care after surgery, and no common complications such as wound infections or convulsions occurred. The recorded recovery videos can be found in the Supplementary Material S5 (Supporting Information).

## Supporting information



Supporting Information

Supporting Information

## Data Availability

The data that support the findings of this study are available on request from the corresponding author. The data are not publicly available due to privacy or ethical restrictions.
