# Supplementary material for: A Myelin Debris Cleaner for Spinal Cord Injury Recovery: Polycaprolactone / Cell Membrane Assembled Scaffolds
Source: Adv Sci (Weinh). 2025 Jun 26;12(36):e03269. doi: 10.1002/advs.202503269 (PMC12463047; doi:10.1002/advs.202503269)
Supplement: Supplementary file 1 — Supporting Information [file ADVS-12-e03269-s002.docx]

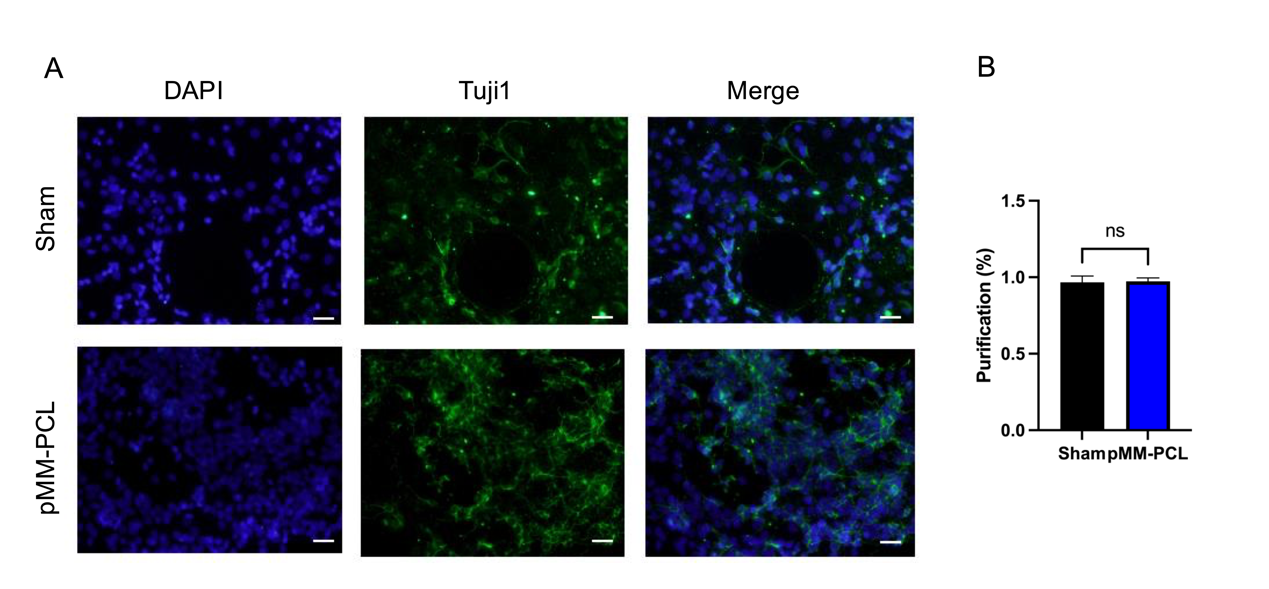


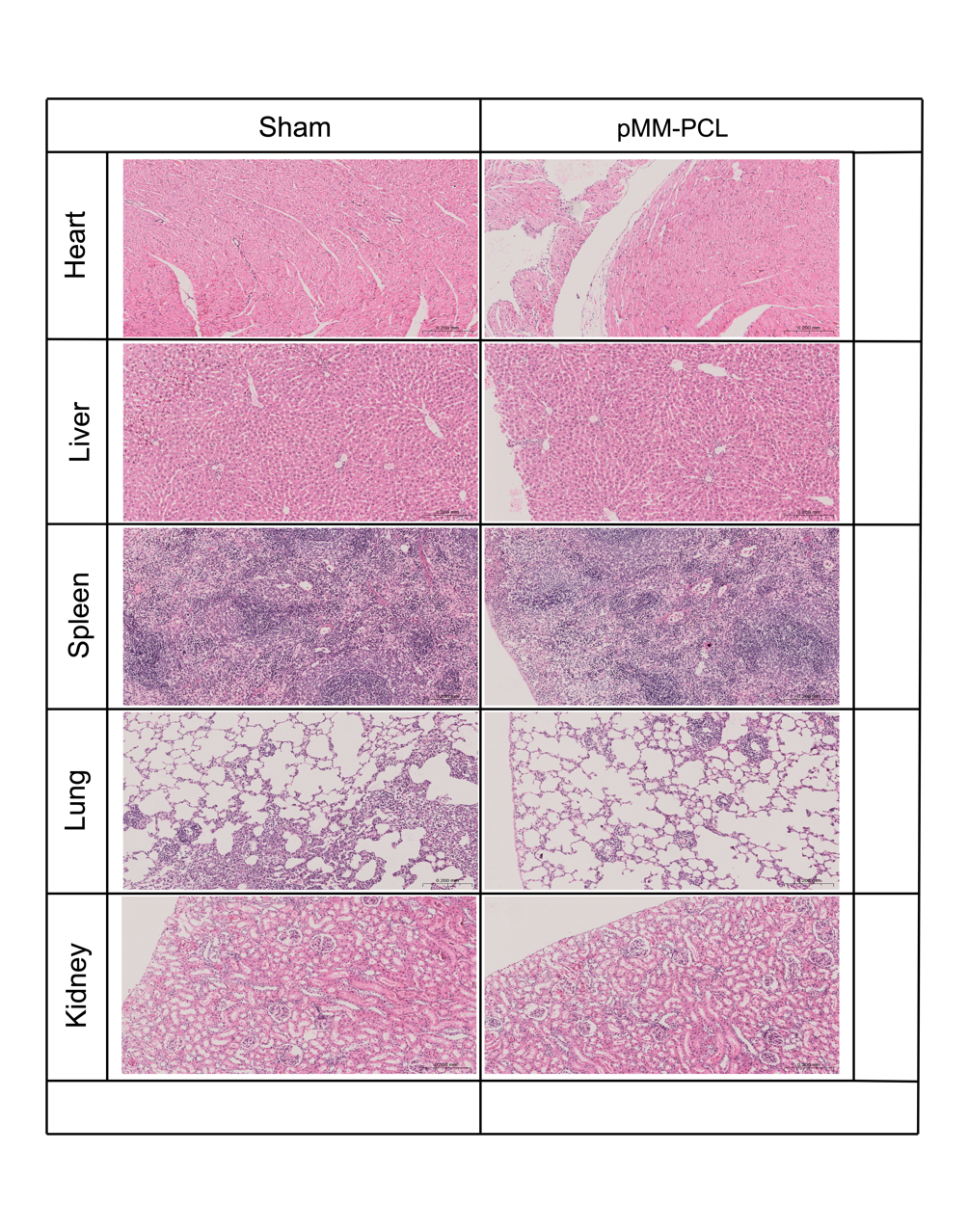
Determination of Neurotoxicity of Composite Materials (A) Tuji1 IF intensity in the Sham and pMM-PCL groups. (B). Statistics analysis of Tuji1 IF intensity in the Sham and pMM-PCL groups (n=3), assessed via t-test. ns=no significance.

Determination of biotoxicity of pMM-PCL.

Supplementary Material 2

Lipidomics

**Chemical reagents and antibodies:** Methyl alcohol, isopropanol and acetonitrile were purchased form Fisher, USA. Cholamine was purchased from China National Pharmaceutical Group Corporation, China. Cholic acid-D4 and D-luciferin free acid were purchased from Shanghai yuanye Biotechnology, China. Chloroform was purchased from Greagent, China.

1. Pretreatment: After weighing 20 mg sample, 267 μL methanol-water (V:V=1:1, containing mixed internal standard, 4 μg/mL) was added, two small steel balls were added, placed at -20℃ for 2min to precool, and then added to the grinder (45 Hz, 2min). Then 267 μL chloroform was added, vortexed for 30 s, extracted by ultrasound for 10 min, left at -20℃ overnight, centrifuged for 10 min (12000 rpm, 4℃), and 200 μL of the lower chloroform layer was loaded into the liquid chromatography-mass spectrometry analysis (LC-MS) injection vial and dried. The lipid residue in the injection vial was redissolved with 134 μL isopropanol-methanol (V:V=1:1), vortexed for 30 s, and sonicated with ice water for 3 min. The solution was transferred to a 1.5 mL centrifuge tube. After centrifugation for 10 min (12000 rpm, 4℃), 100 μL of the supernatant was loaded into an LC-MS injection vial with a lined tube for LC-MS analysis. Quality control samples (QCS) were prepared by mixing the extracts of all samples in equal volumes, and the volume of each QC was the same as the sample. Remarks: All extraction reagents were precooled at -20 ° C before use.
2. LC-MS: The analytical instrument in this experiment was an ACQUITY UPLC I-Class plus liquid chromatography-mass spectrometry system.

Chromatographic condition: Column temperature: 55℃; Mobile phase A: acetonitrile: water =6:4(v/v, containing 10mM ammonium acetate); Mobile phase B: isopropanol: acetonitrile = 9:1 (v/v, containing 10mM ammonium acetate); Flow rate: 0.26 mL/min; Injection volume: 3 μL. Gradient of elution are as follows:

| Time (min) | Flow [ml/min] | Solvent B (%) |
| --- | --- | --- |
| 0 | 0.26 | 32 |
| 1.5 | 0.26 | 32 |
| 15.5 | 0.26 | 85 |
| 15.6 | 0.26 | 97 |
| 18 | 0.26 | 97 |
| 18.1 | 0.26 | 32 |
| 20 | 0.26 | 32 |

Mass spectrum conditions:

| Parameter | Postive ion | Negative ion |
| --- | --- | --- |
| Spray Voltage (V) | 3500 | -3000 |
| Capillary Temperature (°C) | 300 | 300 |
| Aux gas heater temperature (℃) | 350 | 350 |
| Sheath Gas Flow Rate (Arb) | 45 | 45 |
| Aux gas flow rate (Arb) | 10 | 10 |
| S-lens RF level | 50 | 50 |
| Mass range (m/z) | 150-1500 | 150-1500 |
| Full ms resolution | 70000 | 70000 |
| MS/MS resolution | 17500 | 17500 |
| NCE/stepped NCE | 25, 35, 45 | 25, 35, 45 |

Proteomics

**Chemical reagents and antibodies:** iRT standard peptide was purchased from Biognosys, Switzerland. Lysates, PMSF, and phosphatase inhibitors were purchased from Beyotime, China. LDS Sample Buffer, 4X and Dry powder of electrophoresis buffer was purchased from Tanon, China. Precast gel and eStain LG Protein Staining and Decoloring Concentrate Kit were purchased from GenScript, USA. Molecular weight markers of unstained proteins was purchased from Thermo Scientific, USA. DTT was purchased from Adamas-beta, British. IAA, NaCl, Tris-HCl (pH6.8 pH8.8) were purchased from Sangon, China. NH4HCO3 was purchased from Shanghai yuanye Biotechnology, China. Chromatographic grade acetone, formic acid and ammonium acetate were purchased from CNW, China. Trypsin was purchased from Helios, China. EDTA-2Na and Tris saturated phenol were purchased from Solarbio, China.

The total protein was extracted from the spinal cord samples, one part of which was used for protein concentration determination and SDS-PAGE detection, and the other part was used for trypsin digestion. After removing salt from the enzymatic peptide, the samples were identified by LC-MS/MS. For LC-MS/MS identification, DIA technology was used to collect the mass spectrum data of each sample for spectral matching, extraction of quantitative information, and subsequent statistical analysis.

1. Protein extraction: Liquid nitrogen was added in spinal cord samples, and thoroughly ground. An appropriate amount of sample powder was removed and transferred to a 1.5mL centrifuge tube /2mL high-speed centrifuge tube. 800ul of phenol extract was added, phosphatase inhibitor and protease inhibitor PMSF were added to bring the final concentration to 1mM. The cold grinder was ground at -35°C, 60 Hz, 120s and repeated once. An equal volume of phenol-Tris-hcl (7.8) saturated solution was added and mixed at 4°C for 40 minutes, during which time the mixture was mixed with multiple shaking. The phenol upper layer was collected by centrifugation at 7100rpm for 10 min at 4°C. Five times the volume of a precooled 0.1M ammonium acetate-methanol solution was added and precipitated overnight at -40°C. The precipitate was collected by centrifugation at 12000rpm for 10 min at 4°C. Five times the volume of precooled methanol was added for cleaning and mixed slightly. The precipitate was collected by centrifugation at 12000rpm for 10 min at 4°C and repeated once. Step 9,10 was repeated twice with acetone instead of methanol to adequately remove the methanol. The precipitate was collected by centrifugation at 12000rpm for 10 min at 4°C. After drying at room temperature (generally about 5 min), the solution was dissolved in sample lysate and dissolved at room temperature for 3-5 min. The solution was centrifuged at 12000rpm for 10min at 4 ° C, the supernatant was removed, and the supernatant was centrifuged again. The supernatant was the total protein solution of the sample, which was determined for protein concentration and then stored at -80°C for later use.
2. Determination of protein concentration

According to the instructions of the BCA kit, the desired volume of chromogenesis solution was configured according to buffer A: Buffer B=50:1(v/v). Part of the protein solution to be tested was removed and diluted with ultrapure water (to prevent the concentration from being too high and beyond the working range of the standard curve). A clean 96-well plate was prepared and the following gradient of BSA standard protein solution was added: 0,1,2,4,8,12,16,20 μL, and then the corresponding volume of ultrapure water was added to each well to replenish the volume to 20μL. Two μL of the protein solution to be tested was added to the 96-well plate, and three multiple Wells were set for each sample, and the volume was similarly supplemented to 20μL. Add 200μL of pre-configured chromogenic solution to each well (the chromogenic solution must be used on the spot!) The reaction was carried out at 37 ° C for 30min. A microplate reader was used to measure the absorbance value (wavelength 562nm). The standard curve was calculated according to the known concentration and absorbance value of the standard protein solution, and the protein concentration value was calculated by substitution of the absorbance value of the sample to be tested.

1. Sds-polyacrylamide gel electrophoresis

An appropriate amount of protein from each sample was separated by 12% SDS-PAGE. The separated gels were stained with Coomassie brilliant blue using an eStain LG protein stainer. The stained gels were imaged using an automatic digital gel image analysis system.

1. Enzymolysis by trypsin

According to the measured protein concentration, an appropriate amount of protein was taken from each sample, and the different groups of samples were diluted with lysates to adjust to the same concentration and volume. DTT was added to the above protein solution so that the final concentration of DTT was 5 mM, mixed, and incubated at 55°C for 30min. Cooling was carried out on ice until room temperature was reached. The corresponding volume of iodoacetamide was added so that the final concentration was 10 mM, thoroughly mixed, and left in the dark at room temperature for 15min. The protein was precipitated by adding 6 times the volume of acetone to the above solution and left at -20 ° C for more than 4 hours or overnight. The precipitate was collected by centrifugation at 8000×g for 10 min at 4°C and acetone was volatilized for 2-3 min. The precipitate was redissolved by adding 50mM NH4HCO3, and 1mg/ml Trypsin/mL of 1/50 sample mass was added and digested overnight at 37 ° C. The enzymatic hydrolysis was terminated by adding phosphoric acid to adjust the PH value to about 3.

1. Salt removal from the enzymatic peptide

Activation: The column was activated with 200 μL methanol and repeated twice for a total of three times. Equilibration: The column was activated with 200 μL of equilibration solution (0.1% formic acid, 100% water) and repeated twice for a total of three times. Load sample: Volume of 50-500μL sample, adjust the vacuum, and keep the droplet speed at 1 mL/min (about 1 drop/second) and repeat the load once. Wash: Wash with 200μL of 0.1% formic acid in water, repeated twice, for a total of 3 times. Elution: Peptides were eluted with 150μL 50% acetonitrile-water (containing 0.1% formic acid), repeated 2 times for a total of 3 times to obtain 450μL of eluate, which was dried by vacuum waving.

1. Detection by LC-MS/MS high resolution mass spectrometry

Before mass spectrometry injection, each sample was mixed according to the volume ratio iRT: sample to be tested =1:20 and used as an internal standard. iRT standard (Biognosys, ThermoFisher) is a hybrid kit containing 11 synthetic peptides that do not exist in nature. Its stability, sensitivity and retention time have been optimized. It is relatively stable and does not affect the test sample.

DIA chromatographic conditions

| Time | Gradient | Flow rate |
| --- | --- | --- |
| 0 | 7.5%B | 0.7 |
| 8 | 35%B | 0.7 |
| 9 | 100%B | 0.7 |
| 10 | 100%B | 0.7 |

DIA mass spectrometry conditions

| Items | Para. |
| --- | --- |
| Capillary | 1.6KV |
| Dry Temperature | 180℃ |
| Dry Gas | 3.2 L/min |
| Mass Range | 300-1500 m/z |
| Ion Mobility | 0.7-1.3 |
| Collision Energy | 20-59 eV |
| Ramp Time | 50ms |

1. PASER software was used to combine all the mass spectrum data and complete the database search of DIA mass spectrum data and protein DIA quantitative analysis. The sequence file of the database search was uniprot-Rattus norvegicus-10116-2024.2.1.

| Item | Value |
| --- | --- |
| Enzyme | Trypsin |
| Max Missed Cleavages | 1 |
| Fixed modifications | Carbamidomethyl (C) |
| Variable modifications | Oxidation (M), Acetyl (Protein N-term) |
| Precursor FDR | 0.01 |
| Precursor Mass Tolerance | 15ppm |

Supplementary Material 4

Myelin Debris Extraction

1. Prepare 10 mL of 100 mM Na₂EDTA solution: Dissolve 0.372 g of Na₂EDTA in 10 mL of ddH₂O.

2. Prepare 500 mL of diluted Tris-Cl buffer: Mix 480 mL of ddH₂O, 10 mL of 1M Tris-Cl, and 10 mL of the prepared Na₂EDTA solution.

3. Prepare 200 mL of 1 M sucrose solution: Add 100 mL of Tris-Cl buffer to 68.46 g of sucrose, then use Tris-Cl buffer to make up the volume to 200 mL.

4. Prepare 150 mL of 0.32 M sterile sucrose solution: Combine 48 mL of 1 M sucrose solution and 102 mL of Tris-Cl buffer. Filter the solution through a syringe filter and dispense it into sterile 50-mL centrifuge tubes.

5. Prepare 100 mL of 0.83 M sterile sucrose solution: Mix 83 mL of 1 M sucrose solution with 17 mL of Tris-Cl buffer. Filter the solution through a syringe filter and dispense it into sterile 50-mL centrifuge tubes.

6. Prepare 100 mL of sterile Tris-Cl buffer: Filter 100 mL of Tris-Cl buffer through a syringe filter and dispense it into sterile 50-mL centrifuge tubes.

7. Sacrifice 10-20 8-12-week-old C57 mice by cervical dislocation. Immerse the sacrificed mice completely in alcohol for 15 minutes, and then decapitate the mice.

8. Take a 10-cm culture dish, pipette 10 mL of Tris-Cl buffer into the dish, and place it on ice for pre-cooling.

9. Use sterile surgical scissors and hemostatic forceps to bluntly dissect and remove the mouse brain. Rinse the brain in the dish containing Tris-Cl buffer to remove bloodstains, cranial bone fragments, hairs, and other impurities as much as possible.

10. Transfer the brain to a 50-mL sterile centrifuge tube and add 0.32 M sucrose solution to make up the volume to 25 mL.

11. Use a sterile handheld rotary homogenizer to disrupt the brain fragments for 2-3 minutes to obtain tissue homogenate. Immediately place the homogenate on ice for freezing or store it in a -20°C refrigerator.

12. Add 3 mL of 0.83 M sucrose solution to each of 8 thin-walled polypropylene ultracentrifuge tubes.

13. Gently layer the tissue homogenate on top of the 0.83 M sucrose solution, taking care not to mix the two layers (especially when adding the first 1 mL of 0.83 M sucrose solution, add it slowly to prevent mixing of the upper and lower layers).

14. Place the 8 ultracentrifuge tubes on a balance with an accuracy of 0.01 g for balancing. If there is a mass difference among the tubes, use the remaining 1 mL of tissue homogenate to adjust the mass of each centrifuge tube until they are balanced.

15. Select an appropriate pre-cooled ultracentrifuge rotor, and centrifuge at 100,000 g at 4°C for 45 minutes (the actual centrifugation time is 45 minutes, including the acceleration and deceleration periods. Set the centrifuge for 54 minutes). Set the acceleration and deceleration of the rotor to the minimum value (centrifuge parameter is 8) to reduce the loss of myelin debris.

16. Carefully and slowly collect the white myelin debris from the interface between the two sucrose densities. Note: The myelin debris are located at the interface between the two sucrose solutions with different densities and appear as white granules.

17. Combine the myelin debris into a 50-mL sterile centrifuge tube and use Tris-Cl buffer to adjust the volume to approximately 20-21 mL.

18. Use a sterile handheld rotary homogenizer to homogenize the myelin debris for 60 seconds.

19. Evenly distribute the myelin debris suspension into 4 clean ultracentrifuge tubes, with 5 mL of suspension in each tube.

20. Place the 4 ultracentrifuge tubes on a balance with an accuracy of 0.01 g for balancing. If the masses of the tubes are different, use Tris-Cl buffer to balance the mass of each centrifuge tube.

21. Select an appropriate pre-cooled ultracentrifuge rotor, and centrifuge at 100,000 g at 4°C for 45 minutes (the actual centrifugation time is 45 minutes, including the acceleration and deceleration periods. Set the centrifuge for 48 minutes). Set the acceleration and deceleration of the rotor to their maximum values (centrifuge parameter is 1).

22. After centrifugation, a white solid precipitate can be observed. Discard the supernatant, add 2 mL of Tris-Cl buffer to each ultracentrifuge tube to resuspend the precipitate, and then combine the suspensions from the 4 ultracentrifuge tubes into 2 tubes, with 4 mL of suspension in each tube.

23. Place the 2 ultracentrifuge tubes on a balance with an accuracy of 0.01 g for balancing. If there is a mass difference among the tubes, use Tris-Cl buffer to adjust the mass of each centrifuge tube.

24. Select an appropriate pre-cooled ultracentrifuge rotor, and centrifuge at 100,000 g at 4°C for 45 minutes (the actual centrifugation time is 45 minutes, including the acceleration and deceleration periods. Set the centrifuge for 48 minutes). Set the acceleration and deceleration of the rotor to their maximum values (centrifuge parameter is 1).

25. Discard the supernatant and resuspend the precipitate in 1 mL of sterile PBS.

26. Take 2 sterile 1.5-mL EP tubes, weigh and record the net weight of the EP tubes on a balance with an accuracy of 0.01 g.

27. Transfer the suspension from the 2 ultracentrifuge tubes into the 2 sterile EP tubes, and centrifuge at 22,000 g at 4°C for 10 minutes.

28. Discard the supernatant, weigh the EP tubes with the myelin debris precipitate again on a balance with an accuracy of 0.01 g. Subtract the previously recorded net weight of the EP tube from this weight to determine the net weight of the myelin debris precipitate.

29. Calculate the amount of PBS to be added based on the net weight of the myelin debris precipitate, resuspend the precipitate, and finally obtain a myelin debris solution with a final concentration of 100 mg/mL. The myelin debris can be stored at -80°C for 6 months.


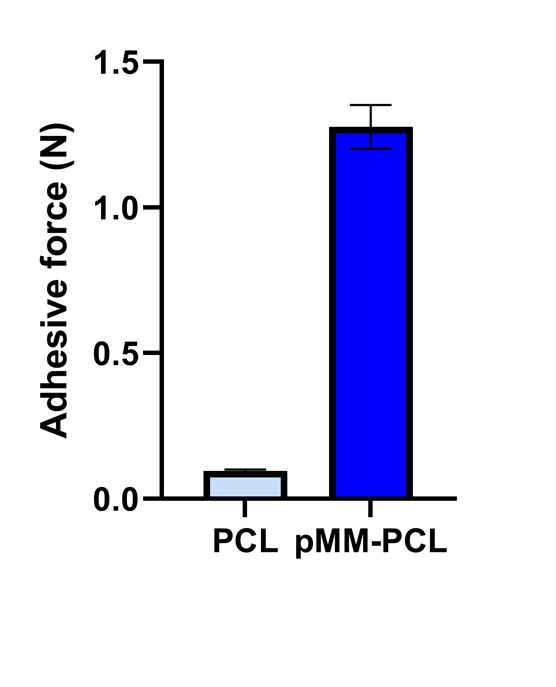


The test of adhesive force of pMM-PCL and PCL to spinal cord tissue.

**Discussion:**

As is shown in the figure, pMM-PCL boasts the more excellent adhesion to spinal cord tissue (from 0.09527 ± 0.00482 N to 1.2769 ± 0.075 N) than PCL. Thus, the feature of robust adhesive force to spinal cord tissue could effectively contribute to pMM-PCL’s lasting biofunction on the surface of the injured site.
