# Supplementary material for: A Myelin Debris Cleaner for Spinal Cord Injury Recovery: Polycaprolactone / Cell Membrane Assembled Scaffolds
Source: Adv Sci (Weinh). 2025 Jun 26;12(36):e03269. doi: 10.1002/advs.202503269 (PMC12463047; doi:10.1002/advs.202503269)
Supplement: Supplementary file 2 — Supporting Information [file ADVS-12-e03269-s001.zip › Supplementary Material 5.zip!/pc12τ╗åΦâ₧Θë┤σ«ÜΣ╣a.pdf]

PC-12(High differentiation)细胞种属鉴定报告

一、 检材：

实验室检测编号为“PC-H2024032633”，细胞名称:PC-12(High differentiation)细胞。

鉴定该株细胞种属,是否有其他物种DNA污染。

二、 检材处理和检验方法：

提取该样本DNA，取适量该样本DNA用种属荧光复合引物MIX对动物线粒体COI基因进行扩增（参考文献:Species identification in cell culture: a two-pronged molecular approach,In Vitro Cellular & Developmental—Animal (2007),扩增产物在智阅基因分析仪GenReader 7010上进行毛细管电泳检测和分析。

三、 检验结果：

实验中阴性及阳性对照结果均正确。

上述细胞株的毛细管电泳检测图谱见附图。

四、 分析说明：

该细胞DNA扩增后图谱清晰，结果良好：内参基因位置，扩增峰正常；

大鼠的种属引物位置出现特异峰，其它种属特异引物位置未见扩增峰。

五、 检验结论：

编号为“PC-H2024032633”的细胞样品为大鼠源细胞，未见其它种属DNA污染。

种属复合扩增MIX表

1-MO	小鼠	10-18S	18S内参	19-CR	中国仓鼠
2-AF	非洲绿猴	11-SS	猪		
3-OR	穴兔	13-OA	绵羊		
4-RA	大鼠	14-CF	狗		
5-HO	人	16-bt	牛		

网站：www.procell.com.cn
电话：400-999-2100
邮箱：techsupport@procell.com.cn
地址：湖北省武汉市高新大道858号生物医药产业园三期C4栋

附图：PC-12(High differentiation)细胞“PC-H2024032633”的COXI基因扩增产物，毛细管电泳图谱。

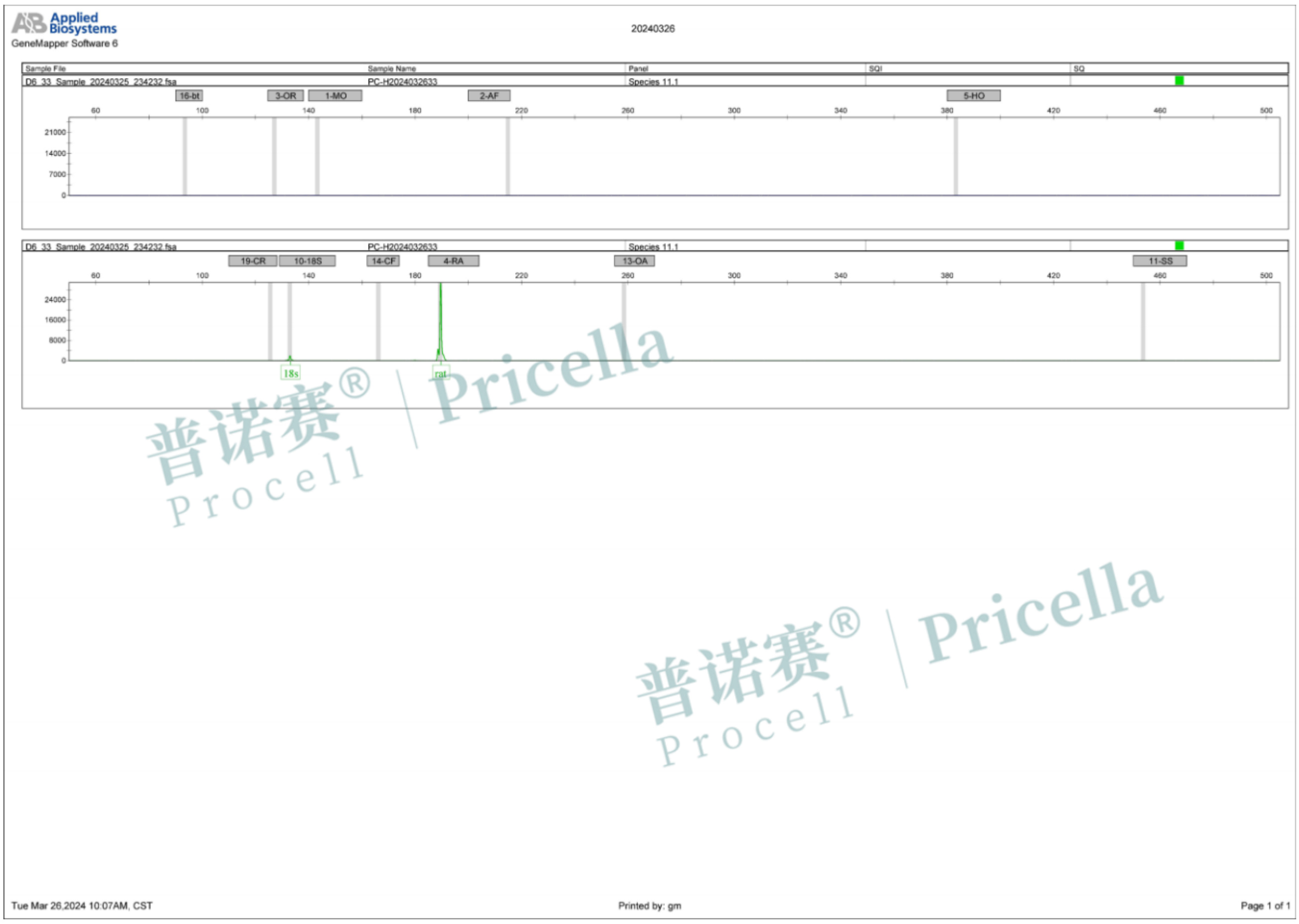

注：内参基因：18S；大鼠源特异引物：4-RA
