# Supplementary material for: A Myelin Debris Cleaner for Spinal Cord Injury Recovery: Polycaprolactone / Cell Membrane Assembled Scaffolds
Source: Adv Sci (Weinh). 2025 Jun 26;12(36):e03269. doi: 10.1002/advs.202503269 (PMC12463047; doi:10.1002/advs.202503269)
Supplement: Supplementary file 2 — Supporting Information [file ADVS-12-e03269-s001.zip › Supplementary Material 5.zip!/rawτ╗åΦâ₧Θë┤σ«ÜΣ╣a.pdf]

附表1：RAW 264.7细胞株PC-H2024012946的STR位点的基因分型结果。

STR Loci	样品名称:RAW 264.7（编号：PC-H2024012946）	数据库名称：RAW 264.7
1-1	16	15,16
1-2	17	17
2-1	16	16
3-2	14	14
4-2	22.3	22.3
5-5	14	14
6-4	17,18	18
6-7	12	12
7-1	25.2	25.2
8-1	13	13
11-2	17	17
12-1	16	16
13-1	16.2	16.2
15-3	22.3	22.3
17-2	14,16	14,16
18-3	18	18
19-2	14	14
X-1	24	24
TH01		
D5S818		
ExPASy数据库匹配度95.00%，匹配位点数18（https://web.expasy.org/cellosaurus-str-search/）		

备注：

1. 根据国际细胞鉴定委员会(ICLAC)制定的细胞 STR 鉴定标准，细胞系的匹配度≥80%时，认为它们具有相关性，即衍生于共同的祖先细胞；匹配度在 55% 至 80% 之间，需要进一步验证相关性；小于 55%，表明两者不具有相关性。
2. 图谱有效峰为真实的 PCR 条带;小峰和非特异性条带在计算中忽略不计。
3. STR 数据比对结果默认 ExPASy，数据来源包括 ATCC，DSMZ，JCRB 等细胞库以及文献和资料记载，数据库入口 https://web.expasy.org/cellosaurus-str-search/。

网站：www.procell.com.cn
电话：400-999-2100；027-87287608
邮箱：sales@procell.com.cn
地址：湖北省武汉市高新大道858号生物医药产业园三期C4栋

普诺赛® Procell | Pricella
武汉普诺赛生命科技有限公司
Procell Life Science&Technology Co.,Ltd.

附图1：RAW 264.7细胞（编号为PC-H2024012946）STR位点的基因分型结果。

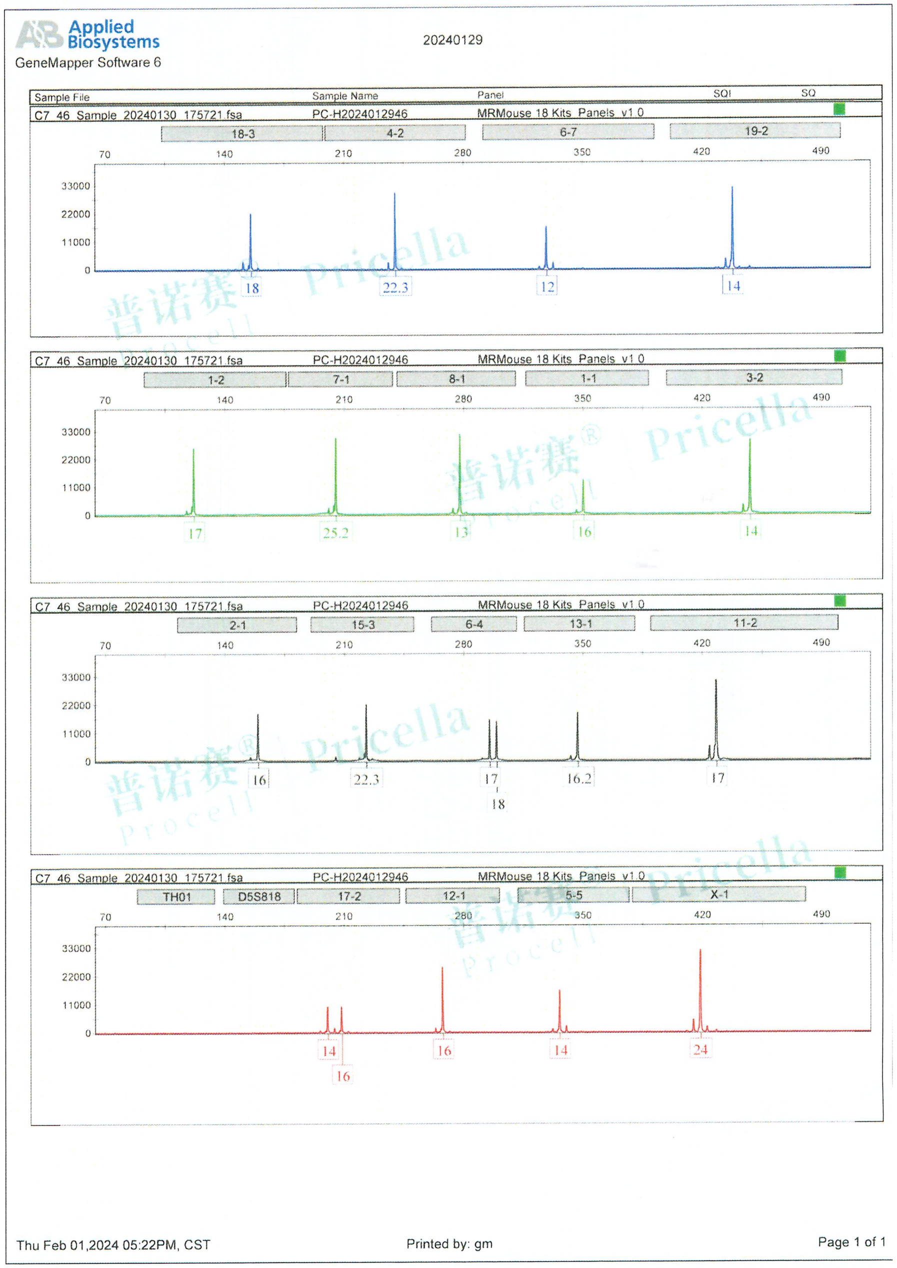

网站：www.procell.com.cn
电话：400-999-2100；027-87287608
邮箱：sales@procell.com.cn
地址：湖北省武汉市高新大道858号生物医药产业园三期C4栋

RAW 264.7细胞STR鉴定报告

一、 材料处理和检验方法

取适量RAW 264.7细胞(编号PC-H2024012946,1×10^{6})使用TIANamp Genomic DNA Kit提取DNA，采用小鼠基因分型试剂盒（Mouse STR v2.0）对18个小鼠位点和TH01、D5S818这2个人源位点进行复合扩增，在智阅基因分析仪GenReader 7010上对扩增产物进行检测，使用GeneMapper Software6软件(Applied Biosystems)对检测结果进行分析，并与ExPASy数据库进行比对。

二、 检测结果

实验中阴性及阳性对照结果均正确。

RAW 264.7 细胞株的 STR 位点的基因分型结果见附表，分型图谱见附图。

三、 分析说明

RAW 264.7 细胞株基因组 DNA 扩增后图谱清晰，分型结果良好。

四、 检验结论

1. RAW 264.7 细胞株 DNA 进行小鼠细胞 STR 分型结果显示，分型结果良好，该细胞株为单一来源小鼠细胞，且没有人源细胞污染。
2. 在 ExPASy 细胞库中找到与其细胞分型 95.00%相匹配的细胞，细胞名称为 RAW 264.7。

网站：www.procell.com.cn
电话：400-999-2100；027-87287608
邮箱：sales@procell.com.cn
地址：湖北省武汉市高新大道858号生物医药产业园三期C4栋

普诺赛® Procell | Pricella

武汉普诺赛生命科技有限公司
Wuhan Pricella Biotechnology Co., Ltd.

Certificate of Analysis

RAW 264.7

货号：CL-0190
报告批准日期:2024-04-22
规格：1×10^6Cells/Vial×2Vials
保存温度:-80℃
批号：L870411020201

Quality Profile

检测项目	单位	标准	结果
外观	/	标签清晰正确、管身外观完好，管盖盖紧	合格
细菌	/	镜检无污染	合格
真菌	/	镜检无污染	合格
支原体	/	阴性	合格
活细胞数	cell	≥1×10^6	合格
活率	/	≥75%	合格
细胞形态	/	不规则形	合格

质检结论：

参考《RAW 264.7细胞产品技术要求》，判定该产品**合格！**

武汉普诺赛生命科技有限公司 质检专用章

报告人：李杰

批准人：熊薇

网站: www.procell.com.cn
电话: 400-999-2100
邮箱: techsupport@procell.com.cn
地址: 湖北省武汉市高新大道858号生物医药产业园三期C4栋
